# Temporal transcriptional dynamics in cutaneous leishmaniasis reveal novel targets for therapeutic interventions in a dermal mouse model

**DOI:** 10.3389/fimmu.2026.1752217

**Published:** 2026-05-12

**Authors:** Jessica Lobo-Silva, Cibele Orge, Almiro Pires da Silva Neto, Bruno Vinagre Ribeiro, Regiane Degan Fávaro, Joyce Karoline da Silva, Sara Patrícia de Oliveira Santos, Sara Nunes, Valdomiro Silveira Moitinho-Junior, Aldina Barral, Natalia Tavares Machado, Pablo Ivan Pereira Ramos, Ricardo Khouri, Leonardo Paiva Farias

**Affiliations:** 1 Laboratório de Medicina e Saúde Pública de Precisão (MeSP2), Instituto Gonçalo Moniz, Fundação Oswaldo Cruz, Salvador, Brazil; 2 Departamento de Ciências da Vida, Universidade do Estado da Bahia, Salvador, Brazil; 3 Escola Bahiana de Medicina e Saúde Pública (EBMSP), Salvador, Brazil; 4 Biotério, Instituto Gonçalo Moniz, Fundação Oswaldo Cruz, Salvador, Brazil; 5 Centro de Integração de Dados e Conhecimento para Saúde, Instituto Gonçalo Moniz, Fundação Oswaldo Cruz, Salvador, Brazil; 6 Patologia, Diagnóstico por Imagem e Medicina Legal (DPML), Universidade Federal da Bahia (UFBA), Salvador, Brazil

**Keywords:** BALB/c mouse model, cutaneous leishmaniasis, immune response, *Leishmania braziliensis*, microRNAs (miRNAs), wound healing, transcriptomics

## Abstract

Cutaneous leishmaniasis (CL) caused by *Leishmania braziliensis* results in chronic skin ulceration and remains challenging to treat. While human transcriptomic studies have identified pathways driving immunopathology, the early events of infection and the molecular transitions from lesion formation to healing are still poorly understood. Here, we performed a longitudinal transcriptomic analysis of skin lesions and draining lymph nodes (dLNs) in the BALB/c ear dermal model infected with *L. braziliensis*, which recapitulates features of human CL. Using bulk RNA sequencing at 2, 6, and 48 hours and at 14, 35, and 77 days post-infection, we characterized differential gene expression, pathway enrichment, and gene co-expression networks. Ulcerated mouse lesions (Day 35) recapitulated 77% of the inflammatory pathways described in human CL, with many persisting at Day 77 despite “clinical healing”. Mice displayed additional upregulation of genes linked to macrophage polarization (*Il12a*, *Il12b*, *Il4*), nitric oxide metabolism (*Arg1*, *Nos2*) and epidermal differentiation (e.g., *Crnn*, *Rptn*, *Tchh*, *Lce* members). Gene co-expression analysis revealed stage-specific gene modules (M) associated with early innate responses (M3), tissue damage (M1), epithelial-mesenchymal transition (M4), and skin barrier remodeling (M6). A long non-coding RNA-enriched module (M2) was selectively downregulated during the ulceration. Cross-species comparison of ulcerated lesions revealed 16 conserved microRNAs and 12 shared epigenetic regulators, including *Mir155*, *Mir142*, *Sp140*, and *Kdm6b*, with known roles in inflammation and tissue repair, representing promising host-directed therapeutic targets. Together, this study provides a comprehensive temporal framework of host responses to *L. braziliensis* and identifies actionable non-coding RNAs and epigenetic pathways with translational potential for CL therapy.

## 1 Introduction

Leishmaniasis comprises a spectrum of diseases caused by more than 20 species of intracellular protozoan parasites of the genus *Leishmania*. Clinical manifestations vary widely depending on parasite species and host immune status (1). Cutaneous leishmaniasis (CL) is the most common form and treatment efficacy remains suboptimal, with cure rates as low as 50% (2). In the Americas, *Leishmania braziliensis* is the principal etiological agent and a major cause of chronic, ulcerative disease (3, 4).

Advances in omics technologies have enabled systems-level investigation of host-*Leishmania* interactions. Transcriptomic studies of human *L. braziliensis* lesions have revealed central immunopathological pathways. Novais et al. described a hypothetical metapathway leading from CD8+ T cell activation and cytolysis to IL-1β production, which drives immune-mediated tissue damage (5). To understand the progression of *L*. *braziliensis* disease, Christensen et al. used RNA sequencing (RNA-seq) to profile early and late human lesions, demonstrating that these clinical stages were not transcriptionally distinct (6). Additional work showed that chronic, treatment-refractory lesions exhibit increased activation of components of the cytolytic machinery (GZMB, PRF1, GNLY) (7). Across these studies, strong interferon-stimulated gene (ISG) and cytolytic programs consistently emerged (5–7) and were later detected systemically in peripheral blood of *L. braziliensis*-infected individuals (8). More recently, alterations in the skin microbiota have also been linked to disease severity (9).

Murine models have provided complementary mechanistic insights. Single-cell RNA sequencing (scRNA-seq) of *L. major* infection in C57BL/6 mice demonstrated macrophage remodeling within ear lesions, along with downregulation of initiation factor-2 (EIF2) and several ribosomal subunits (10, 11). Another scRNA-seq study demonstrated cellular remodeling caused by *L. major*, suggesting that dermis-resident macrophages orchestrate Th2-type immune responses along with innate lymphoid cells and eosinophils within the lesion, serving as a replicative niche for *L. major* during the progression of more severe CL (12).

Despite these advances, our understanding of the temporal sequence of events during *in vivo* infection remains limited, particularly with respect to early host responses, the transition to ulceration, and the dynamics of lesion healing. Furthermore, gaps remain in understanding the communication between the immune responses occurring in the skin and the draining lymph node (dLN). Although the BALB/c ear dermal model of *L. braziliensis* infection has been widely used for nearly two decades—for example, in preclinical vaccine and drug screening studies—and recapitulates key features of human CL, including ulceration, parasite dissemination to lymphoid tissues, and a dominant Th1-type response (13), no longitudinal, genome-wide transcriptomic analysis has been performed in this model.

In parallel, there is growing interest in host-directed therapies (HDTs) to improve clinical outcomes in leishmaniasis. In cutaneous leishmaniasis, particularly in *Leishmania braziliensis* infection, tissue damage and treatment failure are often driven by dysregulated host inflammatory responses rather than parasite burden alone. HDTs aim to modulate host immune, metabolic, or epigenetic pathways rather than directly targeting the parasite, with the potential to mitigate immunopathology, reduce lesion chronicity, and enhance the efficacy of antiparasitic drugs (14, 15). Epigenetic regulators, microRNAs, and inflammatory signaling pathways have emerged as promising intervention points, yet few have been evaluated *in vivo* in the context of *L. braziliensis* infection.

In this study, we conducted a comprehensive time-course RNA-seq analysis of *L. braziliensis* infection in the murine ear, profiling both lesions and matched dLNs from early infection through ulceration and healing. By integrating differential gene expression, pathway enrichment, co-expression networks, and cell-type deconvolution, and by comparing our data with multiple human and murine CL datasets (5–7, 9–11), we define conserved and species-specific molecular programs underlying disease progression. We also identify non-coding RNAs and epigenetic regulators with translational potential as host-directed therapeutic targets.

## 2 Materials and methods

### 2.1 Ethics statement

All procedures involving animals were approved by the Ethics Committee on the Use of Animals (Comissão de Ética no Uso de Animais – CEUA) of the Instituto Gonçalo Moniz (IGM-FIOCRUZ, Salvador, Bahia-Brazil) under license no. 008/2014 and complied with national regulations for laboratory animal welfare. All procedures involving euthanasia were performed in accordance with institutional and national guidelines for the humane treatment of laboratory animals.

### 2.2 Parasites and culture

*L. (Viannia) braziliensis* (MHOM/BR/01/BA788) virulence was maintained through passages in BALB/c mice. Parasites were grown *in vitro* until reaching stationary phase in a B.O.D. incubator at 26 °C in Schneider’s insect medium (Invitrogen) supplemented with 10% inactive Fetal Bovine Serum (FBS) (Gibco), 2 mM L-glutamine, 100 U/ml penicillin and 100 µg/ml streptomycin (all from Invitrogen) (13). Stationary-phase promastigotes were used for infection.

### 2.3 Animals and experimental design

**Figure 1:**
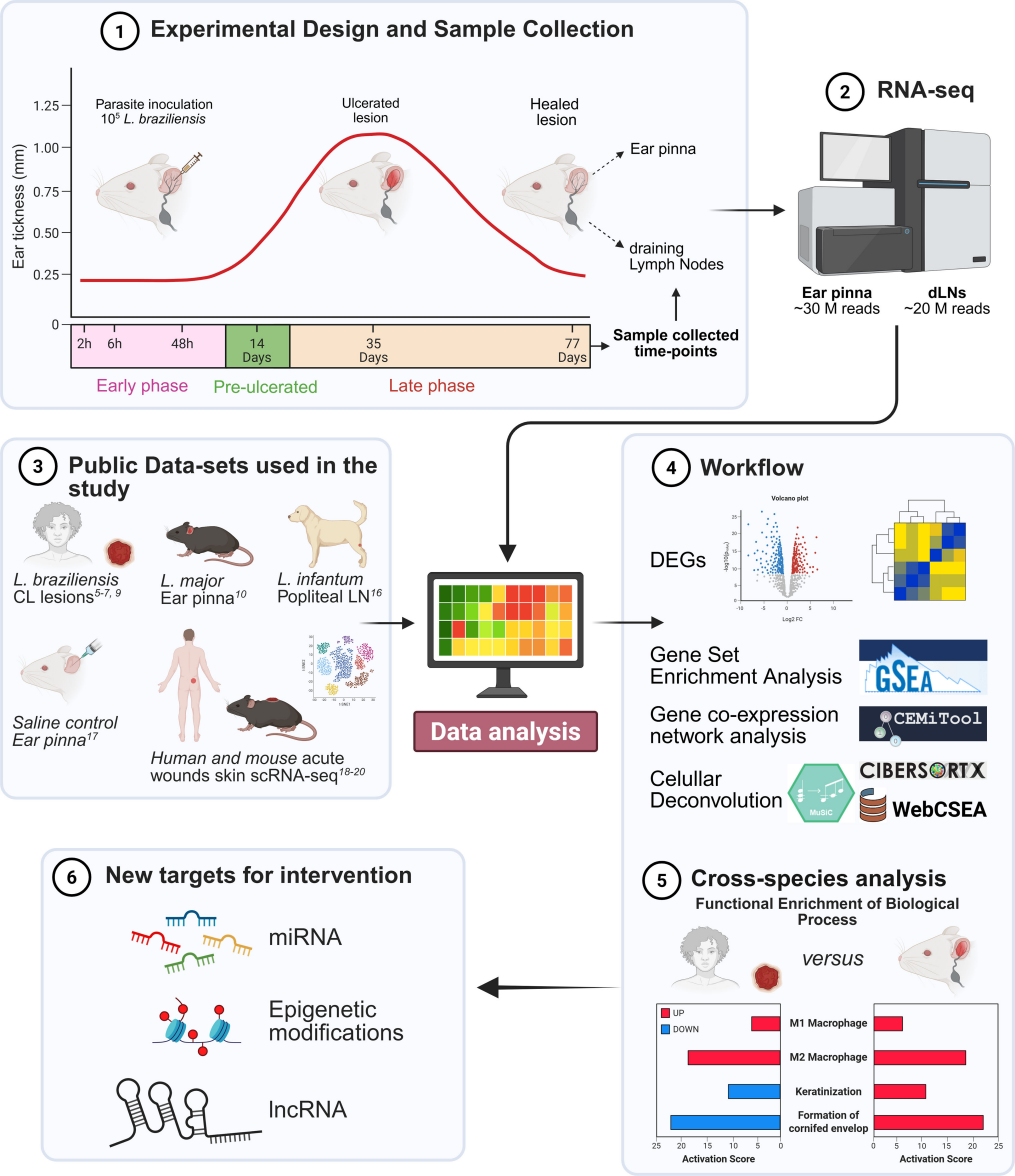
Experimental design and analytical workflow for comparative transcriptomic profiling of *L. braziliensis* infection in mice and humans. (1) BALB/c mice were intradermally inoculated with 10^5^ metacyclic *L. braziliensis* in the ear, and lesion progression was monitored longitudinally by ear thickness measurements. Three mice were euthanized at specified time points (2, 6, and 48 hours, and 14-, 35-, and 77-days post-infection). In parallel, three uninfected control animals were included for comparative analysis. Ear pinnae with lesions and corresponding draining lymph nodes (dLNs) were collected for RNA extraction. (2) RNA sequencing was performed, with a target depth of approximately ~30 million reads per ear sample and ~20 million reads per dLN sample. (3) Additional publicly available datasets were incorporated for comparative analysis (5–7, 9, 10, 16–20). (4) Workflow illustrating the different strategies and tools used for analysis. (5) Cross-species analysis highlighting key transcriptional differences between mice and humans lesions. (6) Identification of conserved targets shared by both species as potential candidates for therapeutic intervention.

Twenty-one female BALB/c mice (6–8 weeks old), were obtained from IGM-FIOCRUZ animal facility. A total of 18 mice were inoculated intradermally in the center of ear pinna with 10^5^ stationary-phase *L. braziliensis* promastigotes in 10 μL of saline using a 27.5-g needle (13); three non-manipulated mice were kept as controls. Ear thickness was recorded weekly with a digital caliper (Thomas Scientific). Mice (n=3 per time point) were euthanized at 2 h, 6 h, 48 h, 14 days, 35 days and 77 days post-infection (dpi) (**Figure 1**). Animals were euthanized using carbon dioxide (CO_2_) inhalation in a dedicated euthanasia chamber with a gradual fill rate of 20% of the chamber volume per minute, with the CO_2_ flow maintained for at least one minute after clinical death, followed by confirmation of death prior to tissue collection. For each animal, the infected ear was collected and immediately snapped frozen and stored in liquid nitrogen until RNA extraction. The retromaxillar lymph nodes draining this ear (dLNs) were collected, fixed overnight in RNAlater solution (Ambion) and then stored in liquid nitrogen until RNA extraction. For clarity, lesions were classified into an early phase (2 h–48 h post-infection, without overt clinical signs), a pre-ulcerative phase (Day 14, characterized by papule formation without tissue breakdown), and a late phase comprising the ulcerated stage (Day 35) and the healed stage (Day 77).

### 2.4 RNA extraction

The cryopreserved ears were processed individually into a fine powder using a porcelain mortar/pestle in the presence of liquid nitrogen. Powdered tissue was homogenized in RLT buffer (Qiagen) for total RNA isolation using the RNeasy Mini kit (Qiagen) following manufacturer’s instructions. To avoid RNA degradation at later time points, tissue was thawed only in the presence of RLT and vigorously vortexed. dLNs were extracted with the RNeasy Micro Kit (Qiagen) following the manufacturer’s instructions. RNA quantity and purity were assessed by NanoDrop 1000 (Thermo Scientific, Waltham, MA) and RNA integrity by Agilent Bioanalyzer (Agilent Technologies, Santa Clara, CA). RNA was frozen at −80 °C until library construction.

### 2.5 Library preparation and sequencing

For ear samples, ribosomal RNA was depleted using the Eukaryote RiboMinus kit (Invitrogen,Carlsbad, CA). Strand-specific libraries were prepared using TruSeq Stranded Total RNA Library Prep workflow (Illumina, San Diego, CA) according to the manufacturer’s recommendations. Libraries were sequenced on an Illumina NextSeq500 system using v2 chemistry in High Output mode, configured for 150 sequencing cycles, generating 75 base pair paired-end reads. For dLNs samples, libraries were prepared using the TruSeq Stranded mRNA Library Prep Kit (Illumina, San Diego, CA) and sequenced on an Illumina HiSeq 2500 system using a stranded paired-end protocol, generating 75 base pair paired-end reads. Target sequencing depth were ~30 M reads per ear sample and ~20 M reads per dLN sample. Library QC metrics and per-sample read counts are provided in **Supplementary Table 1**.

### 2.6 Gene expression data analysis

Read quality control (QC) was assessed using FastQC (http://www.bioinformatics.babraham.ac.uk/projects/fastqc/). Low-quality bases and adapter sequences were removed with Trimmomatic v0.36 (21). Reads passing QC were aligned to the mouse reference genome from GENCODE release M21 (assembly GRCm38.p6) (22) using STAR v2.7.1a with default parameters (23). Host-unmapped reads were subsequently re-mapped to the *L. braziliensis* M2904 genome (24). Annotation (GFF) and sequence (FASTA) files were obtained from the Leish-ESP database (http://leish-esp.cbm.uam.es/). Transcripts counts were generated using featureCounts. The Trimmed Mean of M-values (TMM) normalization was performed to obtain normalization factors across samples and to account for underlying RNA composition (25). Prior to testing for differential expression, low-expression genes were filtered using the default parameters of the *filterByExpr* function implemented in edgeR v. 3.26.8 (26). P-values were corrected for multiple testing using the False Discovery Rate (FDR). Genes were considered differentially expressed (DEGs) when FDR ≤ 0.05 and absolute log2 fold change ≥ 2 for lesion samples or ≥ 1.5 for dLN samples, unless otherwise specified. For comparative analyses, we also incorporated publicly available gene expression datasets from human cutaneous leishmaniasis (CL) lesions, referred to as *Hs* (5), *Hs2* (6), *Hs3* (7), and *Hs4* (9).

### 2.7 Gene set enrichment analysis

To assess genome-wide expression patterns and identify coordinated changes in predefined gene sets across different time points, we performed Gene Set Enrichment Analysis (GSEA, Broad Institute; https://www.broadinstitute.org/) (27). For each time point, genes were ranked by their differential expression p-values, and enrichment was evaluated against curated gene sets. GSEA procedures were carried out as previously described (28).

In addition to our dataset, we applied GSEA to publicly available expression profiles from human CL lesions (*Hs*) (5), *L. major*-induced mouse lesions (*Lm*) (10), and popliteal lymph node aspirates from *L. infantum*–infected dogs (*Li*) (16). To account for nonspecific responses resulting from needle injury and inoculation, we also included a dataset from saline-injected mouse ears (needle control, *NC*) (17). Gene sets used in the analysis consisted of curated canonical pathways from the Kyoto Encyclopedia of Genes and Genomes (KEGG) (29) and the Reactome Pathways Database (30).

### 2.8 Estimation of cell composition

Digital cytometry was performed to characterize the immune infiltrate using the seq-ImmuCC algorithm, which estimates 10 immune cell populations from RNA-seq count data. The method applies least-squares linear regression machine-learning approach to reference signature matrices specifically developed for different mouse tissues. For our analyses, we used the PBMC matrix for skin lesions and the spleen matrix for dLNs (31). Visualizations were generated in R version 3.4.4 (http://www.r-project.org/) using base functions and the ggplot2 package (32). Statistics between groups were assessed by Mann-Whitney U-test using the rstatix package.

To estimate the cell composition from the gene modules identified by CEMiTool (see below), we used the Web-based Cell-type Specific Enrichment Analysis of Genes (WebCSEA) (33) (https://bioinfo.uth.edu/webcsea/). This resource encompasses 111 scRNA-seq panels of human tissues and 1,355 tissue-cell types from 61 tissues across 11 human organ systems. Mouse module genes were first converted to their human orthologs using g:Profiler (https://biit.cs.ut.ee/gprofiler/orth). Enrichment analysis was then performed on WebCSEA, focusing on the top 20 general cell-type datasets. Only cell types with enrichment above the Bonferroni-corrected significance threshold of 3.69 × 10^-5^ (*p*-value set at *α* = 0.05, adjusted for 1,355 comparisons) were displayed.

To identify macrophage subtypes and other cellular populations in these samples—and to compare them with subtypes predicted from bulk RNA-seq data of human CL biopsies (9) - we applied deconvolution techniques using single-cell RNA-seq datasets from murine and human models of acute skin wounds induced by punch biopsy (18–20). A detailed description of all methods can be found in **Supplementary Methods, Appendix**.

### 2.9 Co-expression network and enrichment analysis

To identify groups (i.e., modules) of co-expressed genes in our dataset, we used the Co-Expression Modules identification Tools (CEMiTool) R package, which enables automated module detection through an unsupervised gene filtering method coupled with automated parameter selection and enrichment analysis. Data used in this analysis was normalized as previously described (34). CEMiTool also performs over-representation analysis (ORA) to identify enriched pathways and constructs interaction networks to detect protein-protein interaction (PPI) patterns.

For pathway enrichment, we used the Hallmark gene sets from Mouse Molecular Signatures Database (MSigDB). To identify protein interactions relevant to innate immune responses, we queried the InnateDB database (35). Fisher’s exact test was used to calculate the overlap p-value for each analytic tool; significance was attributed to p‐values < 0.05.

## 3 Results

### 3.1 Gene expression dynamics reveal temporal phases of lesion development and resolution

In the dermal infection model with *L. braziliensis*, ear thickness begins to increase around Day 14 after inoculation, with lesion volume peaking between Days 21 and 35, followed by spontaneous healing around Day 70 (**Figure 1**). Accordingly, sampling time points were selected to capture key phases of infection and lesion evolution, including early host responses, disease progression, ulceration, and resolution. Specifically, early time points (2 h and 6 h) were chosen to reflect immediate innate responses and initial parasite–host interactions, while 48 h captures the transition toward early adaptive immunity phase preceding macroscopic lesion development. Sampling at Day 14 corresponds to the late pre-ulcerative stage, characterized by papule formation. Day 35 represents peak lesion size with ulcerated morphology, whereas Day 77 corresponds to the post-healing phase, marked by lesion resolution. For analytical purposes, we defined the early phase as 2 hours to 48 hours, Day 14 as pre-ulcerative phase, and the late phase as Day 35 (ulcerated lesions) and Day 77 (healed lesions).

A total of 20 ear samples and 21 dLN samples were collected across the following groups: Control, 2h, 6h, 48h, Day 14, Day 35, and Day 77, and analyzed by RNA-seq. Principal component analysis showed that ear samples clustered tightly by time point, indicating a clear temporal pattern in gene expression. Notably, the 48 h time point clustered more closely with control samples (**Supplementary Figure 1A, Appendix**). In contrast, dLN samples exhibited greater variability among biological replicates. Even so, Day 35, corresponding to peak lesion development, showed a distinct transcriptional profile compared to other time points (**Supplementary Figure 1B, Appendix**).

While the sequencing depth was not sufficient for quantitative analysis of *Leishmania* transcript abundance, the proportion of reads mapping to the *L. braziliensis* genome suggests that parasite transcriptional activity was highest at Day 35 in both ear lesions and dLNs (**Supplementary Table 1**, **Supplementary Figures 1C**, **1D, Appendix**).

### 3.2 Lesion analysis

#### 3.2.1 The dermal model recapitulates key inflammatory gene expression patterns observed in human CL biopsies

**Figure 2:**
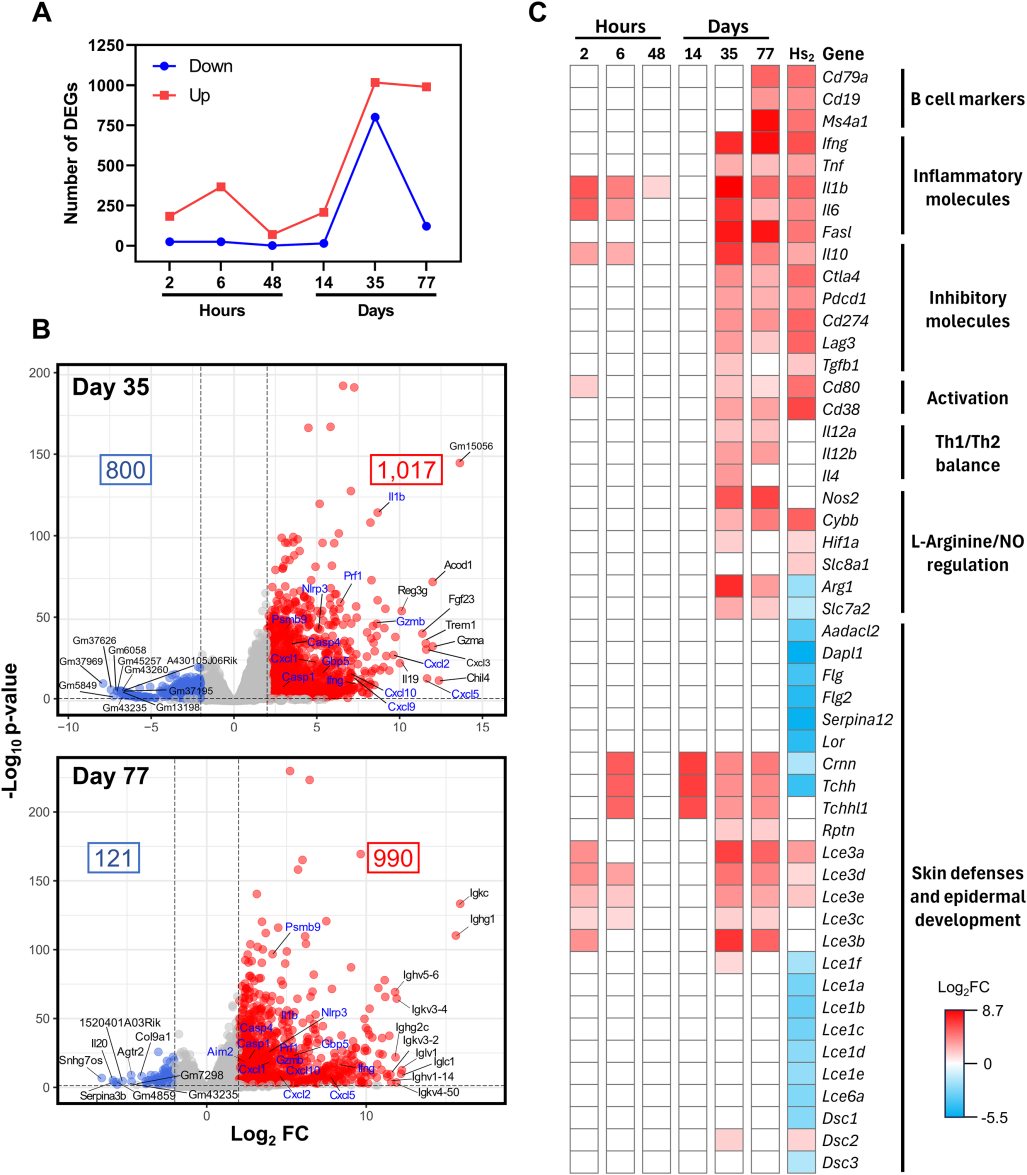
Immune response and epidermal development signatures of lesions of *L. braziliensis* murine infection and human biopsies. **(A)** Number of DEGs identified at various timepoints using FDR< 0.05 and absolute log_2_FC > 2. Fold change was calculated relative to the non-manipulated control group. **(B)** Volcano plots for Day 35 and 77, the most perturbated time points. Red dots indicate upregulated DEGs and blue dots downregulated ones. The number of up- and downregulated genes are shown in the upper right and left corners of each plot, respectively. Genes labeled in blue are linked to the putative metapathway described in (5). **(C)** Heatmap showing the expression profiles of genes related to cell-specific markers, inflammation, immune activation and inhibition, skin defense and epidermal development during *Leishmania* infection in the murine model across various timepoints. For comparison, data from human cutaneous leishmaniasis (CL) biopsies [*Hs_2_*, (6)] are included. Only genes with FDR ≤ 0.05 and absolute log_2_FC ≥ 1.3 were retained for this analysis.

To investigate transcriptomic signatures associated with the course of infection, we performed differential gene expression (DEG) analyses comparing each infected time point to the control group. The transcriptomic profile revealed a peak in DEGs at Day 35 (1,817; 1,017 up, 800 down), followed by Day 77 (1,111; 990 up, 121 down), 6 h (390; 366 up, 24 down), Day 14 (212; 208 up, 14 down), 2 h (207; 183 up, 24 down), and 48 h (70; 69 up, 1 down), with most genes being upregulated (**Figure 2A**). Volcano plots illustrate the magnitude of gene expression changes in infected ears (**Supplementary Figure 2, Appendix**). All DEGs are listed in **Supplementary Table 2**.

Noteworthy, several DEGs identified in the murine model correspond to genes proposed by Novais and colleagues as part of a “metapathway” driving immunopathology in human CL (5), highlighted in blue in **Figure 2B** and in **Supplementary Figure 2, Appendix**. These include immune proteasome components (*Ifng, Tnfa and Psmb9*),chemokines involved in cell recruitment (*Cxcl10, Cxcl9, Cxcl5, Cxcl2, and Cxcl1*),cytotoxicity-associated genes (*Gzmb, Prf1 and Casp4*), and inflammasome-related genes (*Nlrp3, Aim2, Casp1, Il1b, and Gbp5*). Additional genes from this “metapathway” (*Psma4, Psme2, Casp3, Casp7, and Bid*) were also regulated but below the fold-change threshold to be classified as DEGs (**Supplementary Table 3**).

Unexpectedly, most genes within this “metapathway” remained upregulated even in thehealed lesions at Day 77. Furthermore, we did not observe significant inhibition of the seven genesassociated with the skin barrier pathway previously described by Novais et al. (5) (**Supplementary Table 3**).

#### 3.2.2 Divergent immune and skin barrier gene expression between human and mouse lesions

To further investigate the expression of skin barrier-related genes, we expanded our analysis to include key genes identified by Christensen et al., 2016 (*Hs_2_*), who reported the regulation of antimicrobial and skin barrier-associated genes in human CL biopsies. When comparing transcriptional profiles from mice at Days 35 and 77 with those from humans (*Hs_2_*), we observed a notable overlap of B cell markers (*Cd79a, Cd19*, and *Cd20* [*Ms4a1*]) between Day D77 and *Hs_2_* (**Figure 2C**). Inflammatory, inhibitory, and activation markers showed broadly similar expression profiles between mice and humans.

In contrast, mouse lesions at Days 35 and 77 exhibited marked upregulation of key genes involved in macrophage polarization (*Il12a, Il12b*, and *Il4*), as well as genes associated with L-arginine metabolism and nitric oxide production (*Arg1*, *Nos2 and Cat-2 [Slc7a2]*) (**Figure 2C**). Moreover, unlike the human biopsies, where these genes were significantly downregulated relative to healthy skin, mouse lesions showed no evidence of downregulation in skin barrier genes (e.g. *Flg*, *Flg2, Lor, Dsc1)* when compared to non-manipulated controls. Instead, multiple genes from the epidermal differentiation complex (EDC), including *Crnn, Tchh, Tchhl1, Rptn, Lce3a, Lce3d, Lce3e, Lce3c* and *Lce3b*, were significantly upregulated in mouse lesions (**Figure 2C**). A quantitative summary highlighting the contrasting directionality of EDC regulation between species is provided in **Supplementary Figure 3, Appendix**. These findings are further supported by complementary analyses of *Krt* and *Krtp* gene regulation, which are presented in Section 3.2.7 (Gene co-expression analysis during lesion progression and resolution).

#### 3.2.3 The ulcerated and healed phases exhibit the greatest overlap in differentially expressed genes

To identify transcriptional responses associated with each phase of lesion development and the transitions between them, we generated UpSet diagrams from the list of DEGs (both up- and down-regulated) across the sampled time points. This analysis revealed that the largest overlap occurred between Day 35 and Day 77, comprising 406 shared upregulated genes and 57 shared downregulated genes (**Supplementary Figures 4A, B, Appendix**).

We then examined the top 20 DEGs shared across at least two experimental time points. Among the upregulated genes in the lesions, some immunoglobulins transcripts were highly expressed at both Day 35 and Day 77 (**Supplementary Figure 4C, Appendix**). Moreover, key chemokines involved in cell recruitment, such as *Cxcl5, Cxcl3, Cxcl2*, and *Ccl4*, were identified in the intersection of 2h, 6h, Day 35, and Day 77. Notably, several genes previously implicated in leishmaniasis, including *Fpr1, Trem1, Acod1, Gzma, and Olfm4*, were also prominently represented. We further identified four strongly upregulated predicted genes: *Gm4841* (an interferon-inducible GTPase), *Gm15056* (a defensin), and *Gm11555* and *Gm7544* (both keratin-associated proteins).

In contrast, the top 20 downregulated transcripts showed far less overlap across time points, and unlike the upregulated genes, they could not be readily grouped into functional categories. Interestingly, 13 of the downregulated DEGs were computationally predicted genes annotated with the “Gm”prefix, highlighting a predominance of poorly characterized genes among the downregulated DEGs (**Supplementary Figure 4D, Appendix**).

#### 3.2.4 GSEA reveals distinct temporal phases and species-specific immune and epidermal signatures in *L. braziliensis* lesions

**Figure 3:**
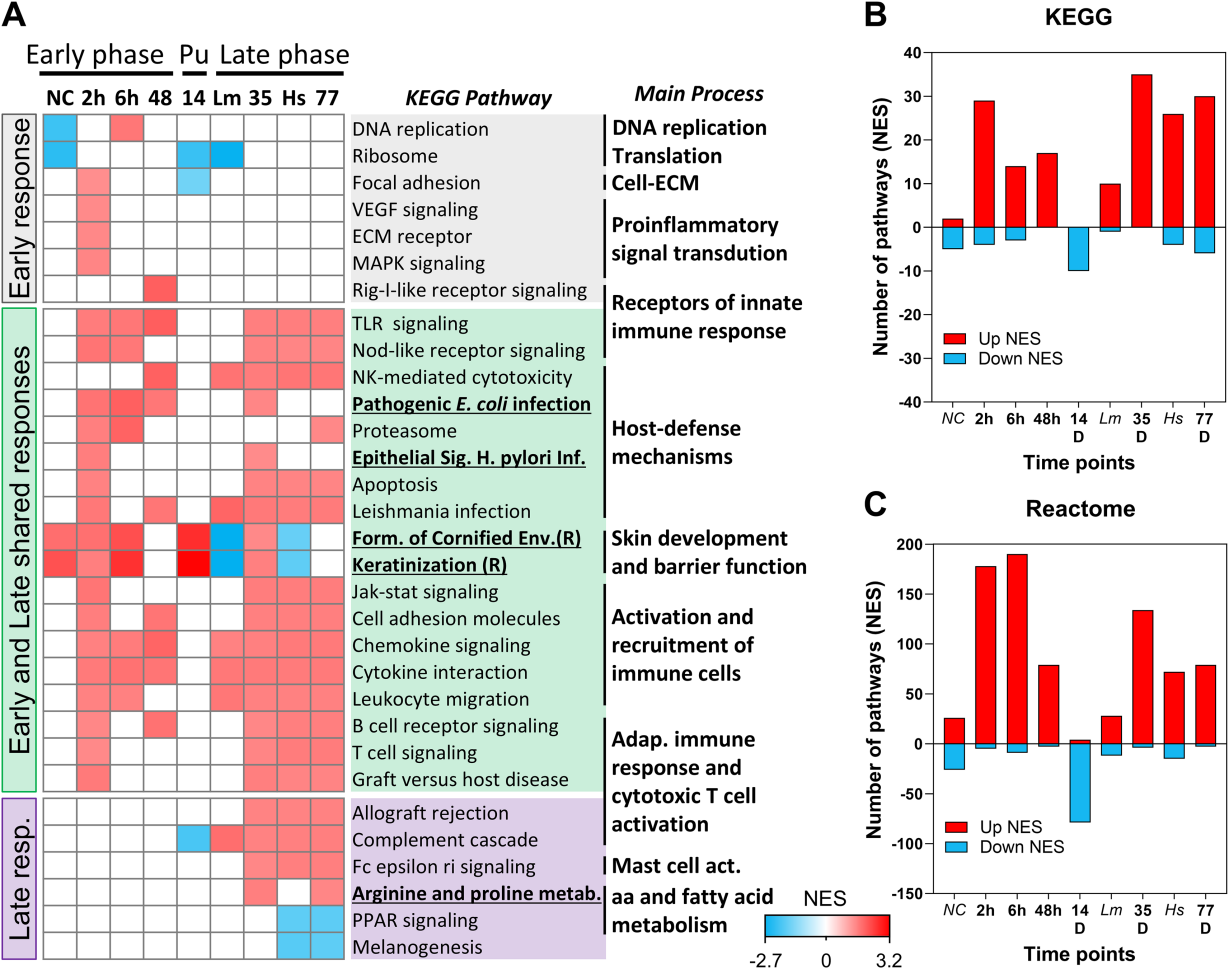
Temporal and cross-species enrichment of immune pathways in leishmania-induced lesions.**(A)** Gene Set Enrichment Analysis (GSEA) showing selected KEGG and Reactome (R) pathwaysenriched in lesion transcriptomes. Columns represent transcriptomic datasets, including intradermal saline injection [needle control, NC; (17)], *L. braziliensis* infection in the BALB/c model (early phase: 2, 6 and 48h; PU (pre-ulcerative phase): Day 14; and late phase: Days 35 (ulcerated) and 77 (healed)], *L. major* infection Day-28 in C57/BL6 mice [*Lm*; (10)], and human CL biopsies caused by *L. braziliensis* [*Hs;* (5)]. Grey shading indicates pathways enriched exclusively in the early response; purple shading highlights those enriched in the late phase; and green shading shows pathways shared across early and late responses. Rows represent pathways, with colors intensity reflecting activation based on normalized enrichment score (NES). Only pathways with FDR ≤ 0.05 were included. Underline pathways indicate contrasting enrichment between murine ulcerate lesions (Day 35) and human CL biopsies. **(B, C)** Total number of KEGG and Reactome pathways with positive or negative NES identified by GSEA, respectively. The complete list of enriched pathways is provided in **Supplementary Tables 4**, **5**.

To gain insight into gene regulation, even at time points with few DEGs, we initially performed KEGG pathway enrichment analyses using the full set of genes detected by RNA-seq in lesion samples. To account for nonspecific responses caused by mechanical stress from needle injury and inoculation, we included a control dataset from mouse ears injected with saline (needle control, *NC*) (17). We also incorporated datasets from human lesions caused by *L. braziliensis* (5) (*Hs*) and from *L. major*-induced CL in C57/BL6 mice, early sampled 28 days post-infection (10) (*Lm*). Following manual curation and removal of redundant or overlapping gene sets, a heatmap based on normalized enrichment scores (NES) revealed gene sets expressed exclusively at the early phase of lesion development, those shared between early and late phases, and others restricted to the late phase (**Figure 3A**). The pre-ulcerative phase at Day 14 displayed a distinct profile.

In the exclusive early response (labeled in grey), ECM receptor, VEGF, and MAPK signaling pathways were activated as early as 2 hours post-infection (**Figure 3A**). Several additional pathways triggered during the early phase (2-48h) were also reactivated later, at Days 35 and 77 (labeled in green), but with greater intensity. These included pathways involved in host defense, immune cell activation, and cell recruitment (e.g., TLR signaling, NK cell cytotoxicity, apoptosis, *Leishmania* infection, leukocyte migration, and B and T cell signaling).

Late-phase pathways (Days 35 and 77) that overlapped with human lesion biopsies (labeled inpurple) were enriched for T cell cytotoxicity, mast-cell activation, suppression of amino acid andfatty acid metabolism, and decreased melanogenesis. Notably, some host defense-related pathways appeared only in late-stage mouse lesions and were not detected in human samples. These included epithelial signaling in *Helicobacter pylori* infection, *Escherichia coli* pathogenicity, proteasome activity, and arginine and proline metabolism. All KEGG-identified pathways are listed in **Supplementary Table 4** and the number of pathways detected at each time point is shown in **Figure 3B**.

To complement the KEGG analysis and reveal additional functional differences, we performed Reactome pathway enrichment analysis. The most striking observation was a broad transcriptional suppression during the pre-ulcerative phase at Day 14 (**Figure 3C**, **Supplementary Table 5**). Although lesions were papular and non-ulcerated at this time point, 88 Reactome and KEGG pathways were downregulated, spanning extracellular matrix remodeling, immune and inflammatory responses, cell signaling and migration, as well as metabolic and mitochondrial programs (**Figures 3B, C**). In total, 92 pathways were grouped into seven central biological programs, as detailed in**Supplementary Table 5** (Classification of pathways into central biological programs).

Notably, ribosomal and translational pathways were also downregulated, suggesting that reduced biosynthetic capacity may underlie this pleiotropic transcriptional repression. In contrast, only four pathways were upregulated, all related to epidermal differentiation and skin barrier maturation. Accordingly, we refer to Day 14 as a pre-ulcerative (transcriptionally silent) phase, reflecting widespread pathway-level suppression despite the presence of early clinical manifestations.

We next examined pathways showing opposite enrichment patterns between mouse ulcerated lesions(Day 35) and human CL biopsies. Among the 351 pathways analyzed, only two displayed contrastingregulation: *Formation of the Cornified Envelope* and *Keratinization*, both upregulated in mouse lesions but downregulated in human samples. Notably, both pathways were among the four already upregulated during the pre-ulcerative (transcriptionally silent) phase at Day 14, highlighting early activation of epidermal differentiation programs in the murine model. All identified pathways are listed in **Supplementary Table 5**.

This analysis highlights four key findings: 1. A distinct pre-ulcerative “transcriptionally silent” phase at Day 14, characterized by marked downregulation of immune, metabolic, signaling, and protein synthesis-related pathways (e.g., Ribosome, Complement, and Focal Adhesion in KEGG), alongside selective upregulation of epidermal development and skin barrier pathways (Reactome); 2. *L. major*-induced lesions at Day 28 (*Lm*) represent a transitional state, with enrichment of NK-cell cytotoxicity, Complement, and *Leishmania* infection pathways; 3. Pathway profiles at Days 35 and 77 were highly similar; and 4. Although mouse and human *L. braziliensis* lesions shared many enriched pathways during the ulcerated phase, mouse lesions showed additional activation of host-defense pathways and consistent upregulation of epidermal development and barrier function pathways—processes that were instead downregulated in human CL.

#### 3.2.5 Macrophages and natural killer cells predominate during the ulcerated phase of the lesion development

**Figure 4:**
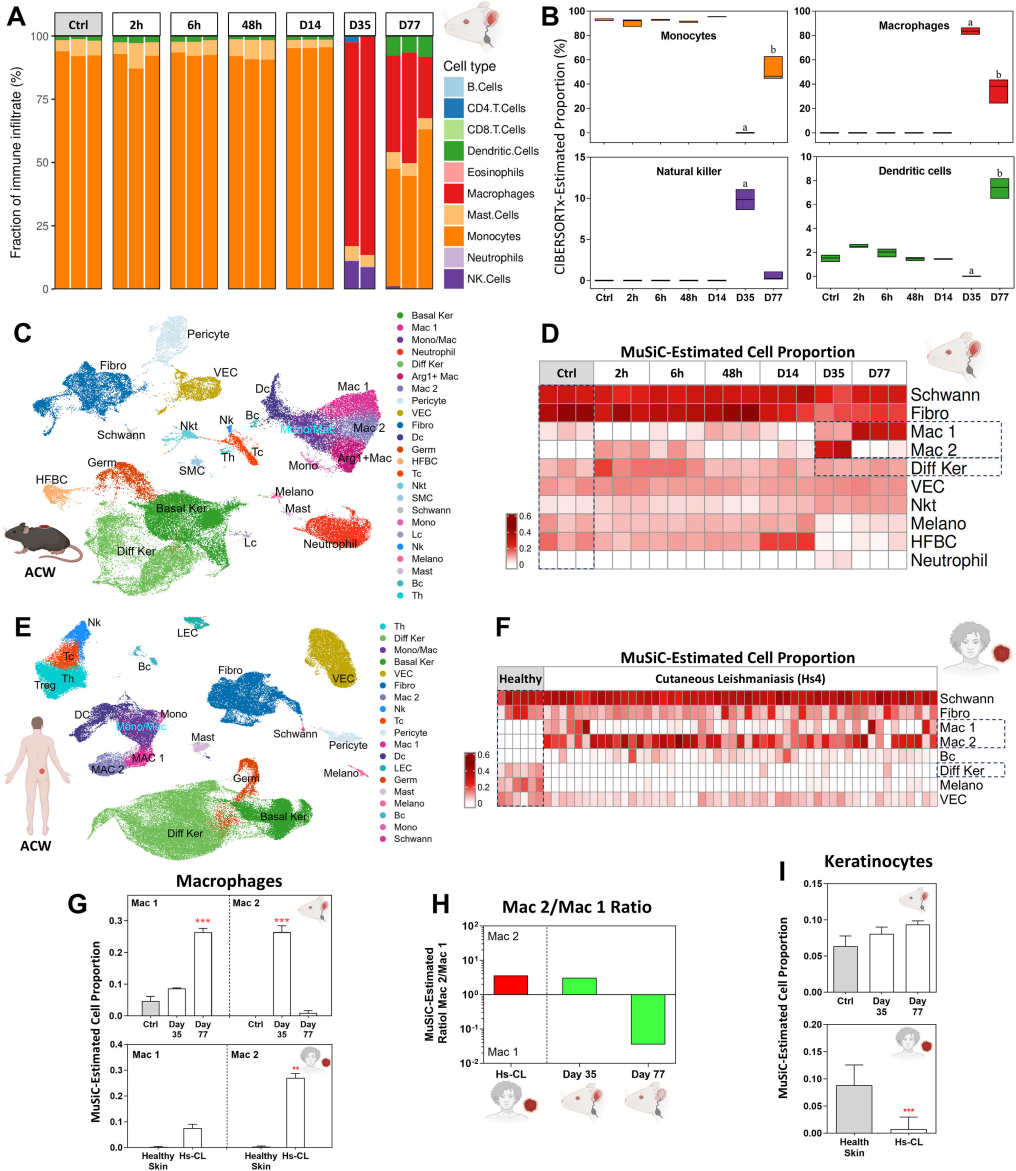
Predicted cell composition for murine and human CL lesion. **(A)** Bar plots showing the distribution of 10 immune cell types in each lesion sample at different time points, as estimated by CIBERSORTx. **(B)** Box plots summarizing the predicted proportions of monocytes, macrophages, natural killer cells and dendritic cells, derived from the data shown in **(A)**. **(C)** UMAP projection of mouse acute wound (mACW) scRNA-seq data. **(D)** Heatmap showing the proportions of major cell clusters identified in each mouse lesion sample across time points, estimated by MuSiC based on the mACW dataset shown in **(C)**. **(E)** UMAP projection of human acute wound (hACW) scRNA-seq data. **(F)** Heatmap showing the proportions of major cell clusters identified in human CL, estimated by MuSiC using hACW dataset shown in **(E)**. **(G, I)** Box plots showing the predicted proportions Mac1 and Mac 2 macrophages and keratinocytes in mouse and human CL samples. **(H)** Mac 2/Mac 1 ratios in mouse and human samples. Significant differences between groups were assessed by unpaired t-test or one-way ANOVA with Tukey’s *post hoc* test. Associated p-values are indicated as **p<0.01 and ***p<0.001.

To estimate immune cell-type composition across lesion time points, we applied CIBERSORTx deconvolution to the bulk RNA-seq data using the ImmuCC PBMC signature matrix, given the absence of a skin-specific matrix. Our initial rationale for using PBMC-derived reference matrix to analyze skin transcriptomic data was to capture circulating immune populations recruited to the skin following infection, while a spleen-derived matrix was the closest available proxy for secondary lymphoid tissue. Most samples showed a predominance of monocytes, likely reflecting the use of a PBMC-derived reference. Significant shifts in immune composition were observed only at Days 35 and 77 (**Figure 4A**).

At Day 35, macrophage abundance increased markedly while monocytes decreased. The sharp rise in natural killer (NK) cells was particularly notable (**Figures 4A, B**). By Day 77, macrophages remained elevated, and dendritic cells also showed a substantial increase (**Figures 4A, B**).

#### 3.2.6 Murine and human lesions exhibit convergent macrophage dynamics and distinct epidermal responses

To further characterize macrophage subtypes and compare cell populations with those in human CL biopsies (9), we performed computational reference-based deconvolution using single-cell RNA-seq datasets derived from murine (**Figure 4C**) and human (**Figure 4E**) acute cutaneous wounds (ACW) generated by punch biopsy. At the time these analyses were performed, no publicly available single-cell RNA-seq datasets from human cutaneous leishmaniasis lesions were available. We therefore opted to use reference datasets derived from acute skin inflammation as a pragmatic approach to enable cross-species comparative analyses. Heatmaps show the estimated proportions of major cell clusters in each sample, inferred using the MuSiC algorithm and the corresponding ACW reference datasets (**Figures 4D, F**). As these reference datasets are derived from acute wound models, the inferred cell-type proportions likely reflect relative functional states rather than absolute cellular identities in chronic leishmaniasis lesions.

In murine lesions, M2-like (regulatory/tissue-adaptive) macrophages were more abundant at Day 35, while M1-like (inflammatory/antigen-processing) macrophages predominated at Day 77 (**Figures 4D, G**). Human CL lesions similarly exhibited a dominant M2-like macrophage profile (**Figures 4F, G**). The M1-like/M2-like ratio for both species is shown in **Figure 4H**; however, the estimated balance between macrophage states may be influenced by differences between acute wound reference datasets and chronic infectious lesions.

Keratinocyte abundance remained relatively stable in mouse lesions compared to controls, with a slight upward trend. In contrast, human CL lesions displayed a clear and substantial reduction in keratinocyte proportions (**Figure 4I**). The full list of identified cell types, estimated cellular proportions, and definingmarker genes is provided in **Supplementary Table 6**.

#### 3.2.7 Gene co-expression analysis during lesion progression and resolution

GSEA evaluates predefined gene sets based on prior biological knowledge (36). In contrast, gene co-expression network analysis identifies modules of genes that share similar expression patterns, allowing phenotype associations without relying on prior assumptions (37). Using CEMiTool, we identified co-expression modules that characterize different stages of lesion progression and resolution.

**Figure 5:**
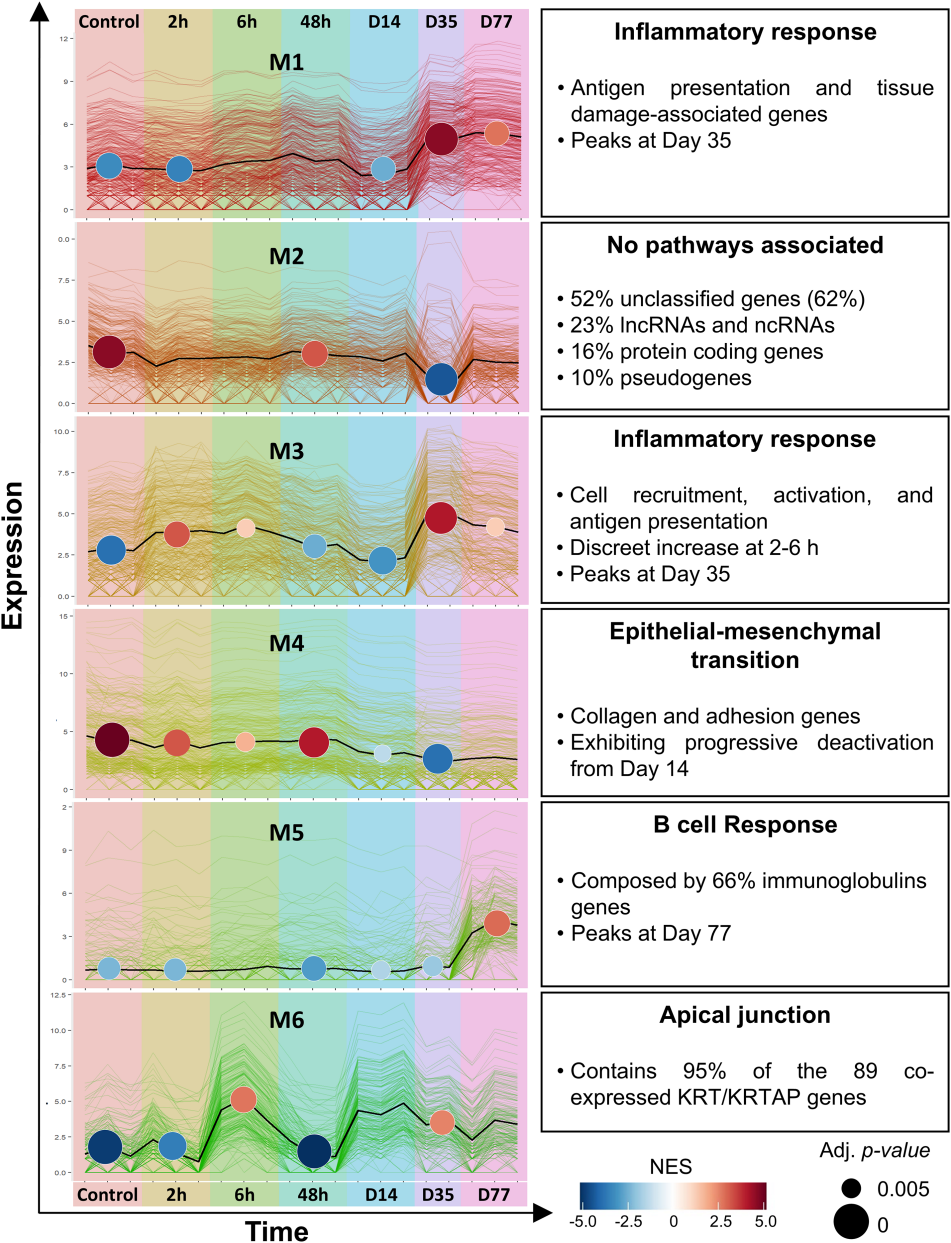
Gene co-expression analysis of murine CL lesions. Six modules (M1–M6) identified with CEMiTool are shown. Colored lines represent individual gene expression profiles, and the black line indicates the module average. Circles show the normalized enrichment score (NES) at each time point; circle color indicates magnitude and direction of enrichment, and size reflects the adjusted p-value. Experimental time points are indicated along the x-axis, and main biological terms identified by over-representation analysis (ORA) are listed to the right of each module, along with key features of interest. Modules and their interaction networks can be explored interactively (38).

Among the eight modules identified, six showed marked variation across the phases of lesion development (**Figure 5**, **Supplementary Table 7**). Over-representation analysis (ORA) revealed that two modules were associated with tissue remodeling (M4 and M6), three with immune pathways (M1, M3 and M5) and one module (M2) could not be assigned any significant process. An interactive exploration of the modules, including interaction networks, is available online (38). To further investigate their functional relevance, we used WebCSEA to infer the predominant cell types associated with each module.

Module M6, associated with apical junction pathways, was the earliest module to show activation. It peaked at 6 hours post-infection and again at Days 14 and 35, coinciding with papule formation and ulcer opening, respectively (**Figure 5**). M6 was enriched for keratin-related genes: 37% of its members were keratins (KRTs) or keratin-associated proteins (KRTAPs). Of 79 co-expressed *Krt/Krtap* genes, 74 were grouped in M6 and were all upregulated during infection (**Supplementary Figure 5A, Appendix**). In contrast, human lesions showed broad downregulation of these genes, with 81% of 43 regulated KRT/KRTAP genes being repressed (**Supplementary Figure 5B, Appendix**). Given the role of keratins in skin structure and repair, this module likely reflects epithelial remodeling during early infection, ulcer formation, and healing.

Module M4, also active during the early stages preceding ulcer opening, was enriched for epithelial–mesenchymal transition and apical junction genes. It includes collagens and adhesion-related genes whose expression declined progressively from early infection through Days 14 and 35 (**Figure 5**). WebCSEA linked M4 to mesenchymal lineages, including fibroblasts, chondrocytes, stromal cells, and other mesenchymal populations, consistent with its structural and matrix-related functions (**Supplementary Figures 6A, B, Appendix**).

Among immune-related modules, M1 and M3 showed similar patterns, with strong upregulation during the ulcerated phase (Days 35–77). M3 also displayed moderate activation early in infection (2–6 h).

Module M1 showed a classic IFN-γ signature, including genes such as *Irf1*, *Irf7*, *Irf8*, *Dhx58*, *Isg15*, and *Stat1*, along with genes linked to antigen presentation (*Ctss*), tissue damage (*Gzmb*), and inflammasome activity (*Casp1*) (**Supplementary Figure 6C, Appendix**). WebCSEA linked M1 to lymphoid populations, including T cells, NK cells, and dendritic cells, highlighting its association with adaptive immune response (**Supplementary Figure 6D, Appendix**).

M3 interaction network, in turn, included key regulators of immune cell recruitment and activation (*Cd14*, *Cd40*, *Vav1*, *Itgb2*, *Ccr1*, *Ccr5*, *Cxcr2*, *Ccl2*, *Nlrp3)* along with cytokine signaling genes (*Socs3*, *Fgr)*, reflecting its role in innate immunity (**Supplementary Figure 6E, Appendix**). WebCSEA linked M3 to monocytes, macrophages, neutrophils, dendritic cells, and myeloid cells (**Supplementary Figure 6F, Appendix**).

Module M5 displayed a distinct late-phase pattern, with marked upregulation at Day 77. WebCSEA linked this module to B cells (**Supplementary Figure 7A, Appendix**). M5 contained 66% immunoglobulin (Ig) genes. Early isotypes (*Ighm*, *Ighd*, and *Ighg1)* were already expressed at Day 35, whereas additional isotypes (*Igha*, *Ighe*, *Ighg2b*, *Ighg2c*, *Ighg3*) were upregulated at Day 77 (**Supplementary Figure 7B, Appendix**). A heatmap of Ig-related DEGs confirmed strong expression in lesion tissues (**Supplementary Figure 7D, Appendix**), and peak expression in dLNs at Day 35 for several isotypes (**Supplementary Figure 7C, Appendix**).

Module M2 exhibited a unique pattern, with downregulation at Day 35 followed by recovery at Day 77. It was not enriched for any known pathways or cell-type signatures, and no coherent functional theme emerged from the interaction network analysis (38). Manual annotation using MGI identifiers revealed that M2 consisted primarily of unclassified genes (52%), non-coding RNAs (23%), pseudogenes (10%), and only 16% protein-coding genes (**Supplementary Figure 8, Appendix**, **Supplementary Table 7**). Notably, most downregulated genes were ncRNAs, pseudogenes, or unclassified elements, whereas upregulated genes were primarily protein-coding. Among them, *Mcpt1, Mcpt2, Mcpt8* (mast-cell proteases) and *Arg1* were notable, though most other upregulated genes lacked clear functional annotation and appeared associated with macrophage or cytotoxic T cell activity.

### 3.3 Draining lymph nodes analysis

To investigate transcriptomic signatures associated with infection dynamics in lymph nodes (dLNs), we applied the same analytical workflow used for lesion samples.

#### 3.3.1 Differentially expressed genes in dLN peak at Day 35 and return to homeostasis by day 77

Gene expression analysis revealed a peak in DEGs on Day 35, with 1,686 genes identified. At this time point, several key components of the immunopathology-associated “metapathway” were highly expressed, including *Il1b, Gzmb, Ifng, Cxcl9, Gbp5, Nlrp3*, and *Cxcl10*. In contrast, only modest transcriptional changes were observed at the other time points, with DEGs ranging from 6 at 6 hours to 67 on Day 14. Remarkably, no DEGs were detected on Day 77, suggesting a return to homeostasis (**Supplementary Figure 9, Appendix**, **Supplementary Table 2**).

Gene expression overlap analysis showed limited sharing of DEGs across time points. The intersections of [Day 14 with Day 35] and [48 hours with Day 35] exhibited the highest numbers of shared upregulated genes (36 and 35 DEGs, respectively) (**Supplementary Figure 10A, Appendix**). The [Day 14-Day 35] intersection also showed the largest overlap of downregulated DEGs (**Supplementary Figure 10B, Appendix**).

We next examined the top 20 DEGs shared across at least two time points. Among these, 12 immunoglobulins genes were upregulated, along with key regulators of B-cell activation and germinal center responses, including *Aicda, Nuggc*, and *Rgs13* (**Supplementary Figure 10C, Appendix**). Conversely, several genes involved in neutrophils and polymorphonuclear leukocytes responses, such as *S100a8, S100a9, Ngp, Mmp8*, and *Camp*, were downregulated, along with genes involved in heme and hemoglobin metabolism (**Supplementary Figure 10D, Appendix**).

#### 3.3.2 Macrophages and B cells are predominant in the dLN during the ulcerative phase

Immune cell populations in the dLN were inferred using CibersortX, following the same approach used for lesion samples, but employing a spleen-derived signature matrix. The most significant changes occurred on Day 35, characterized by increased proportions of macrophages and B cells, and a concurrent decrease in DC and CD4+ T cells (**Supplementary Figure 11, Appendix**). These shifts were consistent with the volcano plot expression patterns and with the top 20 shared DEGs (**Supplementary Figures 9**, **10, Appendix**).

#### 3.3.3 Pathway analysis of immune responses in draining lymph nodes

To identify pathways associated with events preceding ulceration and those occurring during healing, we conducted GSEA on the dLN dataset. Unlike lesion tissue, the dLN transcriptome did not exhibit a clear temporal pattern of immune regulation. Nonetheless, GSEA revealed molecular signatures that were not captured by DEGs analysis alone.

Day 35 was characterized by substantial downregulation of pathways related to signal transduction, cellular differentiation, and proliferation. Interestingly, the antigen presentation pathway was activated as early as 2 hours post-infection and remained active until Day 35. In contrast, apoptosis-related pathways were upregulated only during the healing phase (Day 77) (**Supplementary Figure 12A, Appendix**). Notably, unlike what was observed in lesion tissue, we did not detect a pronounced downregulation of immune-related pathways or protein translation processes in the draining lymph nodes (dLNs) at Day 14.

To contextualize the dLN response, we compared our dataset with a published transcriptomic profile of popliteal lymph nodes aspirate of dogs experimentally infected with *Leishmania infantum* (*Li*), a visceralizing species, sampled 28 days post-infection (16). The comparison revealed only six shared pathways (23%): Lysosome, Chemokine signaling, Proteasome, Antigen presentation, NK-mediated cytotoxicity, and Apoptosis (**Supplementary Figure 12A, Appendix**). Noteworthy, the *Leishmania* infection pathway was absent from the*Li* dataset. All KEGG-identified pathways for the dLN are listed in **Supplementary Table 4**, and the number of pathways detected at each time point is shown in **Supplementary Figure 12B, Appendix**.

Gene co-expression analysis of the dLN did not reveal temporal signatures or enriched pathways as clearly as in the lesions. An interactive analysis of the modules, including interaction networks, is available online (39).

### 3.4 Non-coding RNAs and epigenetic enzymes as potential therapeutic targets

**Figure 6:**
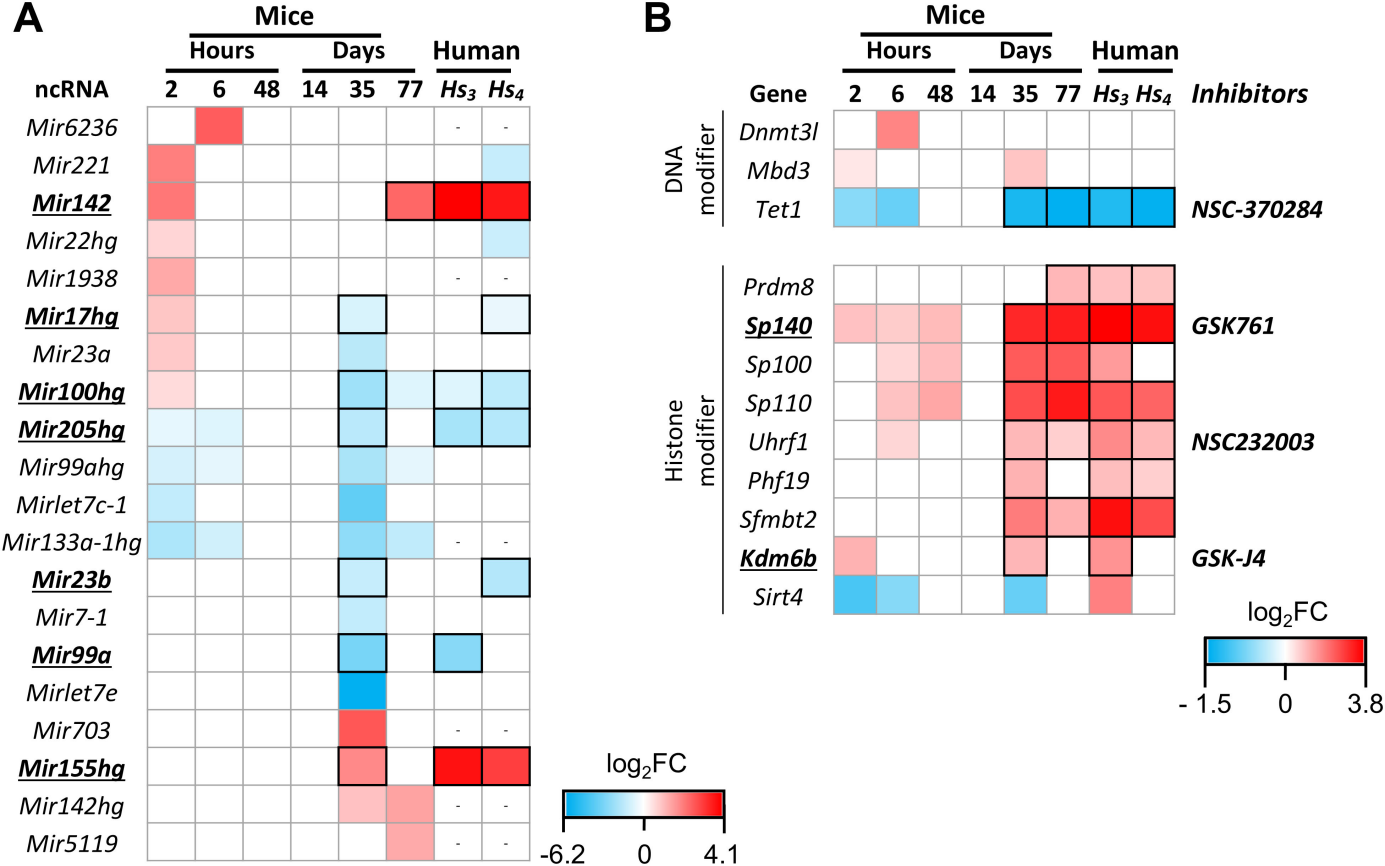
Differential expression of non-coding RNAs and epigenetic enzymes in response to *Leishmania* infection over time. **(A)** Heatmap showing miRNA expression in the murine model of *Leishmania* infection across multiple time points. **(B)** Heatmap of DNA- and histone-modifying enzymes (“Writer,” “Reader,” “Eraser”) with available inhibitors indicated. For comparison, expression data from human cutaneous leishmaniasis (CL) biopsies (7, 9) (*Hs_3_* and *Hs_4_*) are included. miRNAs and epigenetic regulators with conserved regulatory patterns in both species are outlined with a black border on the heatmap, whereas those prioritized based on biological function and/or the availability of inhibitors are underlined. (-) indicates the absence of the corresponding ortholog. Only genes with FDR ≤ 0.055 were included in this analysis.

We next examined specific classes of molecules recently proposed as potential intervention targets, including non-coding RNAs (ncRNAs) and enzymes involved in epigenetic regulation (**Figures 6A, B**). The identification of conserved regulated genes and pathways in both mice and humans reinforces the translational relevance of the murine model for the preclinical evaluation of novel interventions strategies.

Although our study did not employ specific miRNA-capture methodologies, we identified 36 miRNAs-associated transcripts and 8 host genes (mir-HGs) in lesion samples, of which 20 were differentially expressed (**Figure 6A**, **Supplementary Table 8**). Early regulation was evident as soon as 2 hours post-infection, with most miRNAs/mir-HGs upregulated. A second phase of regulation occurred on Day 35, characterized primarily by downregulation.

Several of these miRNAs have been previously implicated in *Leishmania* infection*in vitro* and are known to play crucial roles in parasite persistence, including*Mir221*, *Mir142*, and *Mirlet-7e*, as well as mature miRNAs derived from *Mir205hg*, *Mir133a-1hg*, and *Mir155hg* (40–45) (**Supplementary Table 8**). We also identified six miRNAs not previously associated with *Leishmania* infection: *Mir6236*, *Mir1938*, *Mir17hg*, *Mir23a*, *Mir703*, and *Mir5119* (**Figure 6A**). For cross-species comparison, we incorporated human expression data from Amorim et al. (*Hs3* and *Hs4*) (7, 9). Notably, *Mir142*, *Mir100hg*, *Mir205hg* and *Mir155hg* displayed consistent regulatory patterns across species and datasets, whereas *Mir17hg*, *Mir23b* and *Mir99a* were consistently regulated in at least one dataset (**Figure 6A**).

In case of epigenetic regulators, we analyzed three enzymes classes (46): 18 DNA-modifying enzymes, 103 histone-modifying enzymes, and 7 proteinarginine methyltransferases (**Supplementary Table 9**). Among these, 3 DNA-modifying and 9 histone-modifying enzymes were differentially regulated. Of these, 1 DNA-modifying and 8 histone-modifying enzymes displayed consistent regulation in both mouse and human lesions, with most peaking during the ulcerated phase (**Figure 6B**). Four of these enzymes have available inhibitors or chemical probes, enabling future functional validation.

*Tet1*, a DNA demethylase that contributes to M1 macrophage polarization, was downregulated both at early (2–6 h) and late (Days 35-77) time points, suggesting it is not a suitable target for boosting anti-inflammatory responses. Similarly, UHRF1, an epigenetic repressor of inflammatory genes in myeloid cells, is unlikely to be an optimal inhibition target. In contrast, enzymes linked to inflammatory processes and cytokine production, such as *Sp140* (a bromodomain-containing protein) and *Kdm6b* (a histone demethylase), were positively regulated, and inhibitors like GSK761 and GSK-J4 warrant further functional evaluation.

## 4 Discussion

### 4.1 Mouse lesion closure despite sustained inflammatory response

Several mouse models have been used to dissect the pathogenesis of CL and evaluate potential therapies (47, 48). While lesion kinetics in humans and mice are similar, murine lesions resolve spontaneously within weeks, whereas human healing is markedly slower (13, 49).

To assess how closely the *L. braziliensis* mouse model reflects human disease, we analyzed temporal changes in DEGs, pathways, and co-expression modules. At Day 35, 83% of genes within a “metapathway” linked to CD8^+^ T cell activation and IL-1β production were upregulated, mirroring the immunopathology observed in human CL (5). However, key components of the immunoproteasome (*Psma4, Psme2*) and apoptosis-related genes (*Casp3, Casp7, Bid*) were less modulated, suggesting less sustained CD8^+^ T cell activation during the ulcerated phase in mice. The predominance of *Gzma* over *Gzmb* expression further diverged from the human profile (50–52). We also found no increase of *Prdm1* (Blimp-1) expression in lesions, whereas in C57BL/6 mice, hypoxia in inflamed skin has been shown to induce Blimp-1 during cytolytic CD8^+^ T cell differentiation (53).

**Figure 7:**
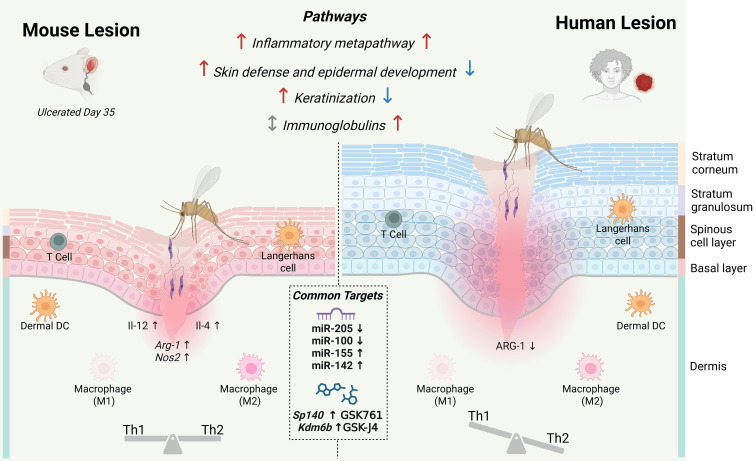
Shared and divergent transcriptional and immunoregulatory programs in *L. braziliensis* lesions from Balb/c mice and humans. Comparative transcriptomic analysis of BALB/c mouse and human lesions revealed a conserved upregulation of inflammatory pathways in both species, while skin defense, epidermal development, and keratinization pathways were upregulated in mice but downregulated in humans. Immunoglobulin-related pathways were selectively upregulated only in human lesions. Macrophage polarization–associated genes (*Il12*, *Il4*, *Arg1*, *Nos2*) were upregulated in murine lesions, whereas only *ARG1* was downregulated in humans. These patterns are summarized by a balanced Th1/Th2 profile in mice and a Th1-skewed response in human lesions, despite comparable M1/M2 macrophage representation. Shared regulation of microRNAs (miR-205, miR-100 ↓; miR-155, miR-142 ↑) and epigenetic regulators (*Sp140*, *Kdm6b* ↑) highlights conserved potential therapeutic targets, including GSK761 and GSK-J4. Symbols indicate direction of regulation: ↑ upregulated, ↓ downregulated and ⇕ not regulated.

Both the ulcerated (Day 35) and healed (Day 77) phases also showed strong upregulation of genes involved in macrophage polarization (*Il12a, Il12b, Il4*) and nitric oxide metabolism (*Arg1, Nos2*) (**Figures 2**, **7**). Despite complete clinical healing by Day 77, many inflammatory pathways remained active, likely reflecting ongoing resolution of inflammation. KEGG analysis revealed a 77% concordance between murine ulcerated lesions and human biopsies. However, mice displayed several additional defense-related pathways and broader macrophage activation signatures, consistent with a more efficient immune response and faster parasite clearance.

Taken together, these data indicate that the mouse model recapitulates key inflammatory features of human CL and mounts a stronger effector response that drives earlier lesion resolution; however, the persistence of inflammatory pathways at Day 77, despite apparent clinical healing, highlights the importance of incorporating molecular endpoints when evaluating therapies in this model.

### 4.2 Divergent epidermal and wound-healing programs in murine and human cutaneous leishmaniasis

Wound healing is essential for maintaining skin integrity and occurs in three interconnected stages: inflammation, proliferation, and tissue remodeling (54). In *L. braziliensis* infection, humans often mount an excessive inflammatory response that contributes to chronic lesions, whereas mice, despite sharing several immunological features with humans, develop a more functional response that allows faster lesion resolution.

Our temporal analysis in mice captured, for the first time in this model, the earliest transcriptional responses to infection (2–48 h and Day 14). A peak of immune activation was already evident at 2 h (Module M3), involving antigen presentation, innate immunity, and leukocyte recruitment (**Figures 3**, **5**, **Supplementary Figure 2, Appendix**). This was followed by the induction of a wound-healing program enriched in keratin and keratin-associated genes (Module M6), which peaked at 6 h and re-emerged at Day 14, preceding ulcer formation (**Figure 5**, **Supplementary Figure 5, Appendix**).

By 48 h, however, most transcriptional modules returned close to baseline levels, resulting in a “silent phase” that persisted until Day 14. PCA, DEG profiles, and GSEA analyses all support the presence of this quiescent window. While the functional consequences of this transcriptional state cannot be directly inferred from the present data, it may reflect a period of limited immune activation that temporally coincides with early parasite persistence, as described in other *Leishmania* infection models (55). At Day 14, the renewed induction of keratin-associated genes (M6) coincided with downregulation of collagen/EMT-related genes (Module M4), potentially priming the tissue for lesion opening (**Figure 5**).

By Day 35, when ulcerated lesions were established, epidermal and skin barrier genes emerged as a major point of divergence between species. In mice, structural proteins such as FLG, FLG2, and LOR, along with stress-response genes including *Crnn* and *Rptn*, which collectively promote keratinocyte differentiation and cornified envelope formation (56–60) were strongly upregulated (**Figure 2**). Likewise, late cornified envelope (LCE) members (*Lce3b, Lce3c, Lce1f*) (61, 62) and desmosome components (*Dsc1-2*), which contribute to repair during inflammation and maintain epidermal cohesion, showed a general pattern of activation in mice but not in human lesions.

Keratinocyte-associated KRT/KRTP genes were broadly and persistently expressed in mice (**Supplementary Figure 5, Appendix**), consistent with recent single-cell studies indicating that murine migratory keratinocytes retain proliferative potential, whereas in humans migratory and proliferative keratinocytes form distinct, non-overlapping populations (20).

Taken together, these findings suggest that mice engage an early compensatory repair program that preserves barrier integrity and supports rapid epidermal closure (63, 64). In contrast, humans fail to sustain equivalent epidermal and barrier gene expression, resulting in progressive barrier deterioration, chronic inflammation, and impaired healing (**Figure 7**).

### 4.3 Comparison with *L. major* lesions reveals common mechanisms of parasite survival

A recent single-cell study of *L. major* lesions in C57BL/6 mice revealed broad transcriptional changes and cellular heterogeneity 28 days post-infection (10). Since this model also develops self-healing, Th1-driven lesions (65), we compared it to our *L. braziliensis* dataset using GSEA (**Figure 3**). The *L. major* Day-28 profile most closely resembled an intermediate stage in the *L. braziliensis* response, lying between Day 14 and Day 35, and all pathways upregulated in *L. major* at Day 28 were enriched in *L. braziliensis* at Day 35.

In contrast, downregulation of Ribosome-related pathways emerged earlier in *L. braziliensis*, during the pre-ulcerative phase at Day 14. Although the functional consequences of this suppression of protein synthesis remain to be experimentally validated, it may reflect a state of reduced host cellular activity that is permissive for parasite persistence during early lesion development, as suggested in other intracellular infection models (66, 67). In support of this interpretation, most pathways at Day 14 were negatively regulated in both KEGG and Reactome databases, with keratinization and cornified envelope formation being the main exceptions (**Figure 3**).

Reduced protein synthesis at this stage may also impair antigen presentation, further dampening host responses (68), although direct functional validation will be required to substantiate this possibility. In line with this interpretation, alterations in host protein synthesis machinery have also been reported in independent *in vivo* models of *Leishmania* infection. Specifically, a study by Venugopal et al. (10) analyzing *L. major* lesions in the ears of C57BL/6 mice at Day 28 post-infection described a coordinated reduction in the expression of multiple ribosomal subunits, accompanied by decreased signaling through the eukaryotic initiation factor 2 (EIF2) pathway. While that analysis was limited to a single experimental time point during the ulcerated phase, our longitudinal analysis of *L. braziliensis* infection in BALB/c mice reveals that the most pronounced inhibition of Ribosome- and Translation-related pathways occurs earlier, at Day 14 post-infection, prior to overt lesion ulceration. At this time point, we observed a broad transcriptional suppression characterized by the downregulation of 88 pathways and the upregulation of only four pathways, consistent with a global reduction in host cellular activity. Together, these findings suggest that suppression of host protein synthesis represents a conserved and temporally regulated feature of *Leishmania* infection, while underscoring the need for additional functional studies to determine whether this transcriptional state reflects active parasite-driven modulation or a host-mediated stress response.

Overall, these cross-species comparisons highlight conserved molecular signatures associated with parasite survival and lesion progression, reinforcing the value of longitudinal and comparative transcriptomic approaches for dissecting host–parasite interactions.

### 4.4 Skin draining lymph nodes and lesions cross talk

The dLN is a key secondary lymphoid organ that initiates Th1 responses following antigen drainage from the skin (65). To date, no transcriptomic study has evaluated the temporal crosstalk between lesions and corresponding dLNs.

Our data indicate that immune responses, and ultimately disease “resolution,” occur more rapidly in the dLN than in the skin. By Day 77, no DEGs were detected in the dLN, and at Day 35, downregulated genes predominated, consistent with a shift toward immune contraction (**Supplementary Figure 9, Appendix**).

Communication between the two tissues is reflected in the coordinated regulation of immunoglobulin expression, peaking at Day 35 in the dLN and at Day 77 in the skin (**Supplementary Figure 7, Appendix**), in line with increasing B-cell frequencies identified by deconvolution analyses (**Supplementary Figures 7A**, **11B, Appendix**). A concurrent reduction in CD4^+^ T cells in the dLN at Day 35 likely reflects trafficking of effector cells to the lesion site (**Supplementary Figure 11, Appendix**).

Some inflammatory markers showed divergent regulation between tissues. For instance, *S100a8* and *S100a9*, along with phagocyte and neutrophil markers, were downregulated in the dLN but upregulated in lesions (**Supplementary Figure 10D, Appendix**). Since these alarmins are important early inflammatory mediators (69) and their absence impairs anti-*Leishmania* responses (70), their downregulation in dLN beginning at Day 14 may reflect either parasite-driven modulation or changes in immune cell composition.

### 4.5 Role of immunoglobulins in lesion resolution in the murine model

In human CL caused by *L. braziliensis*, lesions containing parasite transcripts (PT^Pos^) show elevated B-cell and immunoglobulin expression together with immune inhibitory molecules, suggesting that humoral responses may contribute to parasite persistence (6).

In mice, however, immunoglobulin expression peaks later, at Day 77, when lesions are clinically “resolved” and no active parasite transcription is detected (**Supplementary Figure 1C, Appendix**). This coincides with strong expression of B cell markers (CD79A, CD19, MS4A1) and activation of a dedicated B-cell module (M5) (**Figure 2**, **Supplementary Figure 7**, **Appendix**). By contrast, during the ulcerated phase (Day 35), lesions show intense inflammation and active parasite transcription but low immunoglobulin transcript levels (**Supplementary Figures 1C and 7D, Appendix**).

Together, these findings suggest that in humans, immunoglobulin expression overlaps with parasite persistence, likely reflecting less efficient effector mechanisms, whereas in mice, parasites are cleared before the humoral response peaks (**Figure 7**). Thus, high immunoglobulins levels do not appear to impair lesion resolution in murine *L. braziliensis* model.

### 4.6 ncRNAs and epigenetic regulators as host-directed targets in cutaneous leishmaniasis

A notable finding was the high proportion of downregulated ncRNAs at Day 35, accounting for approximately 26% of all DEGs. Robust ncRNA modulation has also been reported in *in vitro* infection models (71). Several miRNAs detected here, including members of the let-7 family and miR-155, were previously described *in vitro*, and our data now confirm their regulation *in vivo*. In addition, we identified a set of miRNAs, such as miR-23a and miR-99a, as novel candidates linked to *Leishmania* infection (**Figure 6A**).

Among miRNAs consistently regulated in both human and murine ulcerated lesions, therapeutic prioritization must balance the resolution of immunopathology with the preservation of effective microbicidal responses. Within a host-directed therapy framework, we emphasize modulation of excessive inflammation, particularly as these interventions are envisioned as adjuncts to antiparasitic chemotherapy, thereby reducing the risk of uncontrolled *Leishmania* replication. Importantly, the therapeutic impact of HDT modulation is likely to be time-sensitive, with efficacy relying on precise intervention windows rather than on constitutive inhibition or activation.

miR-155 and miR-142, both upregulated and associated with pro-inflammatory immune programs, emerged as compelling but complex candidates for antagomir-based inhibition. miR-155, a key regulator of effector CD8^+^ T cell responses, amplifies cytotoxicity and inflammatory signaling (72) and may contribute to tissue damage in chronic *L. braziliensis* lesions. Its inhibition could therefore theoretically attenuate CD8^+^ T cell driven immunopathology. However, in *L. major* dermal infection model, miR-155 promotes disease susceptibility by favoring Th2 polarization and impairing DC function, while being dispensable for Th1 induction and IFN-γ production by NK and CD8^+^ T cells (45). Whether miR-155 inhibition in *L. braziliensis* would primarily reduce pathogenic inflammation or compromise host defense remains unresolved and requires preclinical evaluation. Similarly, miR-142 has broad immunological roles that warrant careful consideration in therapeutic modulation. It regulates T lymphocytes, NK cells, and dendritic cells, and *mir142* deficiency results in impaired hematopoiesis. miR-142 also supports neutrophil chemotaxis and effective bacterial clearance during skin infection and wound repair (73). Thus, although its inhibition may limit inflammatory cell recruitment and tissue damage, it may also impair protective immune mechanisms, underscoring the need for careful preclinical evaluation in leishmanial disease.

Interpretation of downregulated miRNAs likewise requires caution, as several are transiently suppressed during normal wound repair. The downregulation of miR-99a, miR-100, and miR-23b represents permissive, pro-healing events during the physiological course of tissue repair (74, 75). Accordingly, further suppression of these miRNAs beyond their physiological downregulation may therefore be counterproductive. In the case of miR-205, early studies reported increased expression during the granulation tissue formation phase of cutaneous wound healing (76). However, subsequent *in situ* hybridization analyses revealed strong miR-205 expression in the hyperplastic epidermis surrounding the wound, but reduced levels in the leading migrating keratinocytes of the epithelial tongue (77). While it is tempting to speculate that mimic miR-205 restoration could promote wound repair, its spatially restricted expression suggests cell type–specific functions that cannot be inferred from bulk transcriptomic data alone. Approaches such as *in situ* hybridization or spatial transcriptomics will therefore be essential to accurately define its cellular sources and therapeutic relevance in chronic leishmanial lesions. Together, these miRs candidates highlight mechanistically relevant pathways, but also underscore the challenges of translating them into feasible targets for host-directed therapy in cutaneous leishmaniasis (**Figure 7**).

Epigenetic regulators also emerged as conserved inflammatory mediators. *Sp140* and *Kdm6b* were upregulated in both murine and human lesions, indicating conserved roles in *Leishmania*-induced inflammation (**Figure 6B**). SP140, a bromodomain-containing epigenetic reader, promotes pro-inflammatory transcription and macrophage activation (78, 79). Its inhibitor, GSK761, reduces inflammatory gene expression in macrophages and shows positive effects in Crohn’s disease (80). KDM6B, a lysine demethylase involved in macrophage and DC activation (81, 82), is inhibited by GSK-J4, which reduces cytokine production, including TNF-α, and improves outcomes in models of rheumatoid arthritis, encephalomyelitis and sepsis (83–85). Because epigenetic regulators exert broad transcriptional control, their systemic targeting raises legitimate concerns regarding toxicity, pleiotropy, and cell-type specificity. In the context of cutaneous leishmaniasis, however, these limitations may be mitigated through localized, lesional delivery rather than systemic administration, enabling transient and spatially confined modulation of pathogenic inflammation. Taken together, these results support SP140 and KDM6B as conserved, host-directed targets that could be combined with leishmanicidal drugs to restrain inflammation and improve lesion resolution (**Figure 7**).

Some limitations should be considered when interpreting our findings. Differences in sampling strategy, with human punch biopsies collected at the lesion edge versus whole ear pinna samples in mice, may influence the relative representation of immune and stromal cell populations. In addition, bulk RNA-seq averages transcriptional signals across heterogeneous cell populations, potentially masking cell type–specific or spatially restricted responses that would be better resolved using single-cell or spatial approaches. Reference-based immune cell deconvolution further relies on single-cell datasets derived from acute cutaneous wounds, which may not fully capture the cellular complexity or activation states present in chronic *Leishmania* lesions; accordingly, inferred macrophage populations should be interpreted as relative functional programs rather than fixed polarization states. The modest sample size (n = 3 per time point), together with the lack of orthogonal transcript or protein-level validation, may also limit the detection and confirmation of subtle effects, particularly at early stages. Consequentely, key genes and pathways identified here should be regarded as prioritized candidates for future functional validation.

Finally, as RNA extraction was not optimized for small RNA recovery, the detected miRNA profiles likely represent only a subset of the broader miRNA response and should be considered candidate regulators rather than an exhaustive catalog. Despite these constraints, the strong concordance of transcriptional programs observed in ulcerated lesions across species supports the robustness of the core findings and provides a solid framework for future studies. These include analyses of lesions caused by other *Leishmania* species (e.g., *L. amazonensis*) and the application of single-cell or spatial transcriptomics to resolve lesion architecture and host–parasite interactions in greater detail. Recent applications of spatial transcriptomics to human cutaneous leishmaniasis biopsies (86, 87) represent an important advance, although high-resolution spatial analyses remain limited. Comparative high-resolution spatial transcriptomics of human and mouse lesions would establish a powerful framework to dissect lesion progression and resolution by integrating parasite distribution, cellular composition and molecular signatures.

In summary, our temporal transcriptomics analysis of *L. braziliensis* infection in mice reveals that this model faithfully reproduces the core inflammatory and immunopathological features of human cutaneous leishmaniasis, while mounting a more efficient effector response that accelerates lesion resolution. This response is characterized by the concurrent expression of *Il12* and *Il4*, *Arg1* and *Nos2*, and by higher levels of *Gzma* relative to *Gzmb*. Beyond inflammation, we show that the differential regulation of epidermal and barrier-related genes underlies the divergent kinetics of wound healing between species. Finally, cross-species comparison of ulcerated lesions identified a set of conserved non-coding RNAs and epigenetic regulators—such as miR-155, miR-142, SP140, and KDM6B—that emerge as promising host-directed targets for therapeutic intervention and warrant further functional validation in pre-clinical models.

## Data Availability

The datasets generated for this study can be found in the NCBI’s Sequence Read Archive—SRA (https://www.ncbi.nlm.nih.gov/sra/) repository under BioProject ID PRJNA974386. All other relevant data are within the article and its Supporting Information files.
