# Supplementary material for: Temporal transcriptional dynamics in cutaneous leishmaniasis reveal novel targets for therapeutic interventions in a dermal mouse model
Source: Front Immunol. 2026 May 12;17:1752217. doi: 10.3389/fimmu.2026.1752217 (PMC13201149; doi:10.3389/fimmu.2026.1752217)
Supplement: Supplementary file 1 [file DataSheet1.pdf]

SUPPLEMENTARY MATERIALS

APPENDIX

Contents

1. Supplementary Methods

1.1 Deconvolution using single-cell RNA-seq
- 1.1.1 Processing of Human scRNA-seq Data
- 1.1.2 Processing of Human Bulk RNA-seq Data
- 1.1.3 Processing of Mouse scRNA-seq Data

2. Supplementary Figures

3. References

1. Supplementary Methods

1.1 Deconvolution using single-cell RNA-seq

1.1.1 Processing of Human scRNA-seq Data

Human single-cell RNA-seq datasets were downloaded and analyzed as described by Liu et al. (2025) [1] (accession code: GSE241132) and by Reynolds et al. (2021) [2] (accession code: E-MTAB-8142). Cells were selected based on the following quality control metrics: >300 features (nFeatures), <10% mitochondrial gene content, >500 counts (nCounts), and RNA complexity (nFeature/nCount) > 0.8. The DoubletFinder package (v2.0.6) [3] was used to remove potential doublets. In total, 141,051 cells and 27,636 genes were retained for downstream analysis.

Data normalization and scaling were performed using the Seurat package [4] with default parameters. The top 3,000 most variable genes were used to perform principal component analysis (PCA). For dataset integration, the top 30 principal components (PCs) were used to mitigate batch effects across conditions using the RunHarmony function from the Harmony package [5]. Uniform Manifold Approximation and Projection (UMAP) clustering was generated using the RunUMAP function, after neighbor detection and clustering with FindNeighbors and FindClusters using the Louvain algorithm [6]. Differential expression analysis between clusters was performed using the FindAllMarkers function with the parameters: log2 FC > 1, adjusted p-value < 0.05, and minimum percent > 0.25. Cell clusters (resolution = 0.8) were annotated based on the top marker genes ranked by fold change and informed by annotation provided by Reynolds et al. (2021) [2].

1.1.2 Processing of Human Bulk RNA-seq Data

For bulk RNA-seq analysis, we used the dataset provided by Amorim et al. (2023) [7] (accession code: GSE214397). High-quality reads were filtered using Fastp (v0.23.2) [8] [6] and summarized with MultiQC (v1.0) [9]. Read alignment was performed against the GRCh38.p14 reference genome using STAR (v2.7.10b) with the command line options --runMode genomeGenerate and --sjdbOverhang 99 [10]. Read quantification was carried out using FeatureCounts (v2.0.1) from the Subread package [11]. Differential expression analysis was conducted using the DESeq2 package (v1.42.1) [12].

Bulk RNA-seq data were subsequently deconvoluted using the MuSiC package (v1.0.0), applying the *music_prop* function and retaining only genes present in both datasets [13]. The scRNA-seq data served as a reference to estimate the proportions of different cell types in the bulk RNA-seq samples, using the top 3,000 most variable genes and known cell-type markers.

1.1.3 Processing of Mouse scRNA-seq Data

Mouse scRNA-seq data made available by Cai et al. (2023) [14] (accession code: CRA010641) were retrieved. For this analysis, we selected samples from young mice with lesions at days 0, 2, 4, and 7. Additionally, we included publicly available data from Liu et al. (2024) [1] (accession code: GSE218430), which includes skin samples and one lesion sample from 3 day post-wounding. A total of 86,630 cells and 33,984 genes were retained for further analysis following the same analytical workflow used for the human scRNA-seq datasets. Cell-type annotations were assigned using top-ranked marker genes (by fold change), supported by the annotations provided by Cai et al. (2023) [14].

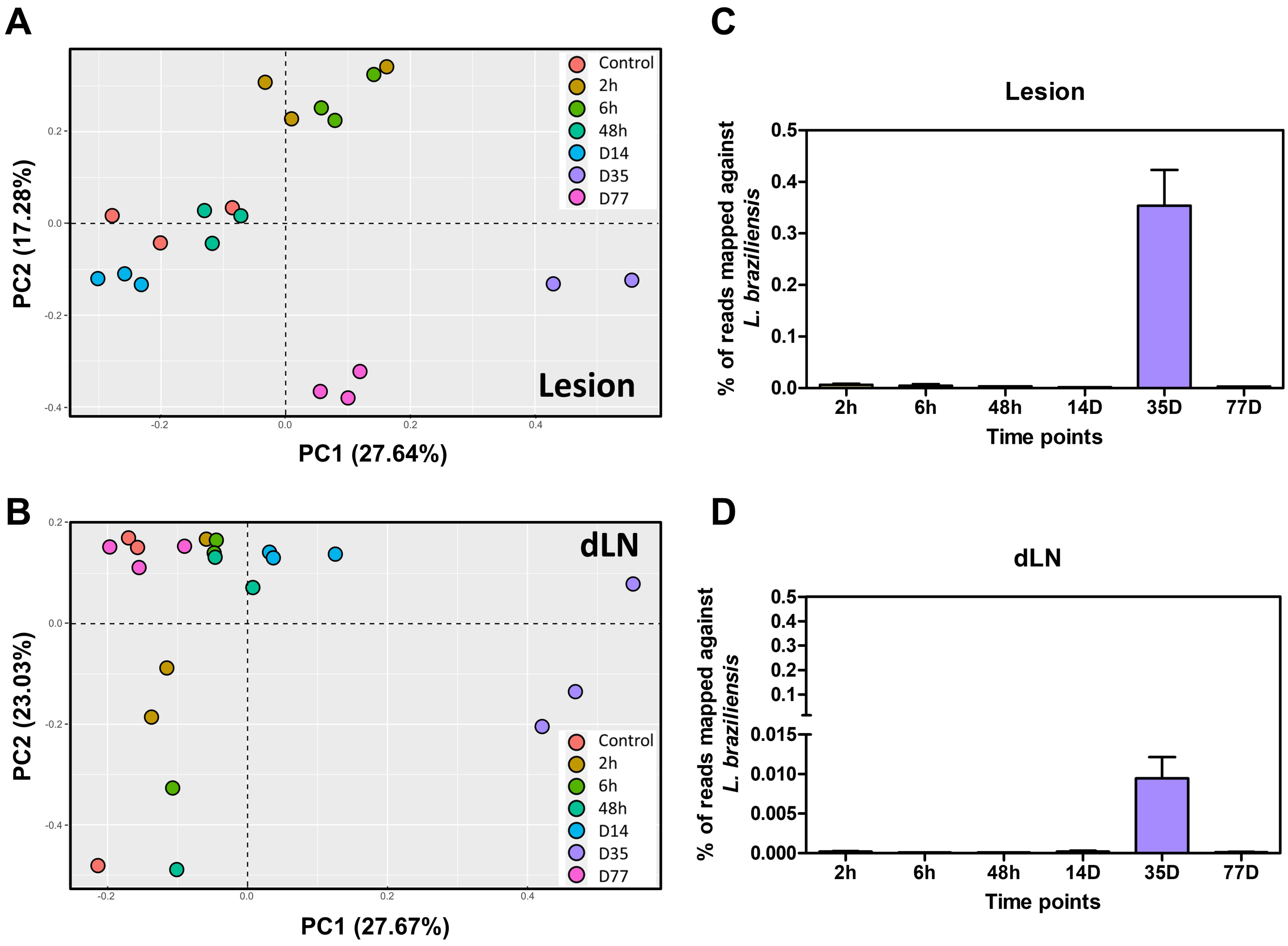

Supplementary Figure 1. Transcriptomic profiling of lesions and draining lymph nodes during murine *Leishmania braziliensis* infection. Principal component analysis (PCA) plots of (A) lesion and (B) draining lymph node (dLN) transcriptomes, with each point representing an individual sample color-coded by time point. Percentages in parentheses indicate the proportion of variance explained by each principal component. (C, D) Percentage of sequencing reads mapped to *L. braziliensis* at each experimental time point in lesions (C) and dLNs (D).

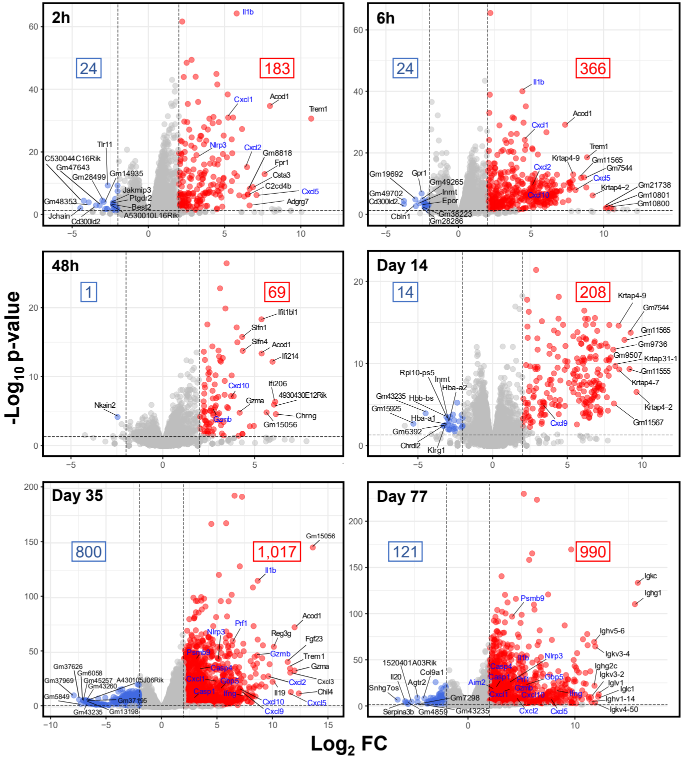

Supplementary Figure 2. Transcriptomic signatures of lesions throughout the course of murine *L. braziliensis* infection. Volcano plots showing differentially expressed genes (DEGs) at 2, 6, and 48 hours, and 14, 35, and 77 days post-infection. Red dots indicate upregulated DEGs ($log_2FC > 2$, FDR < 0.05), and blue dots represent downregulated DEGs ($log_2FC < -2$, FDR < 0.05). Fold changes were calculated relative to the non-manipulated controls. Genes labeled in blue are associated with the putative metapathway described by Novais *et al*. (2015). The number of up- and downregulated genes at each time point is shown in the upper right and upper left corners of each plot, respectively.

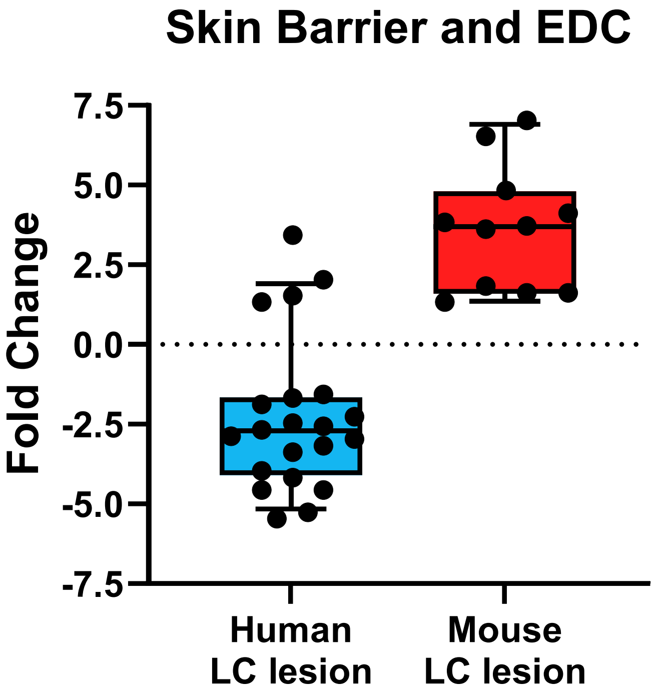

Supplementary Figure 3. Directionality of regulation of skin barrier and epidermal differentiation genes in human and murine cutaneous leishmaniasis lesions. This figure summarizes within-species statistical outcomes and highlights opposing trends in gene regulation between species without performing direct cross-species statistical comparisons. Only genes meeting significance thresholds (FDR $\leq$ 0.05 and $|log_2FC| \geq 1.3$) in at least one dataset were retained for this analysis; therefore, not every human gene is represented by a corresponding murine ortholog in the box plot. Genes and expression values were derived from the datasets presented in the main Figure 2 and include: *Aadacl2, Dapl1, Flg, Flg2, Serpina12, Lor, Crnn, Tchh, Tchhl1, Rptn, Lce3a, Lce3d, Lce3e, Lce3c, Lce3b, Lce1f, Lce1a, Lce1b, Lce1c, Lce1d, Lce1e, Lce6a, Dsc1, Dsc2,* and *Dsc3*.

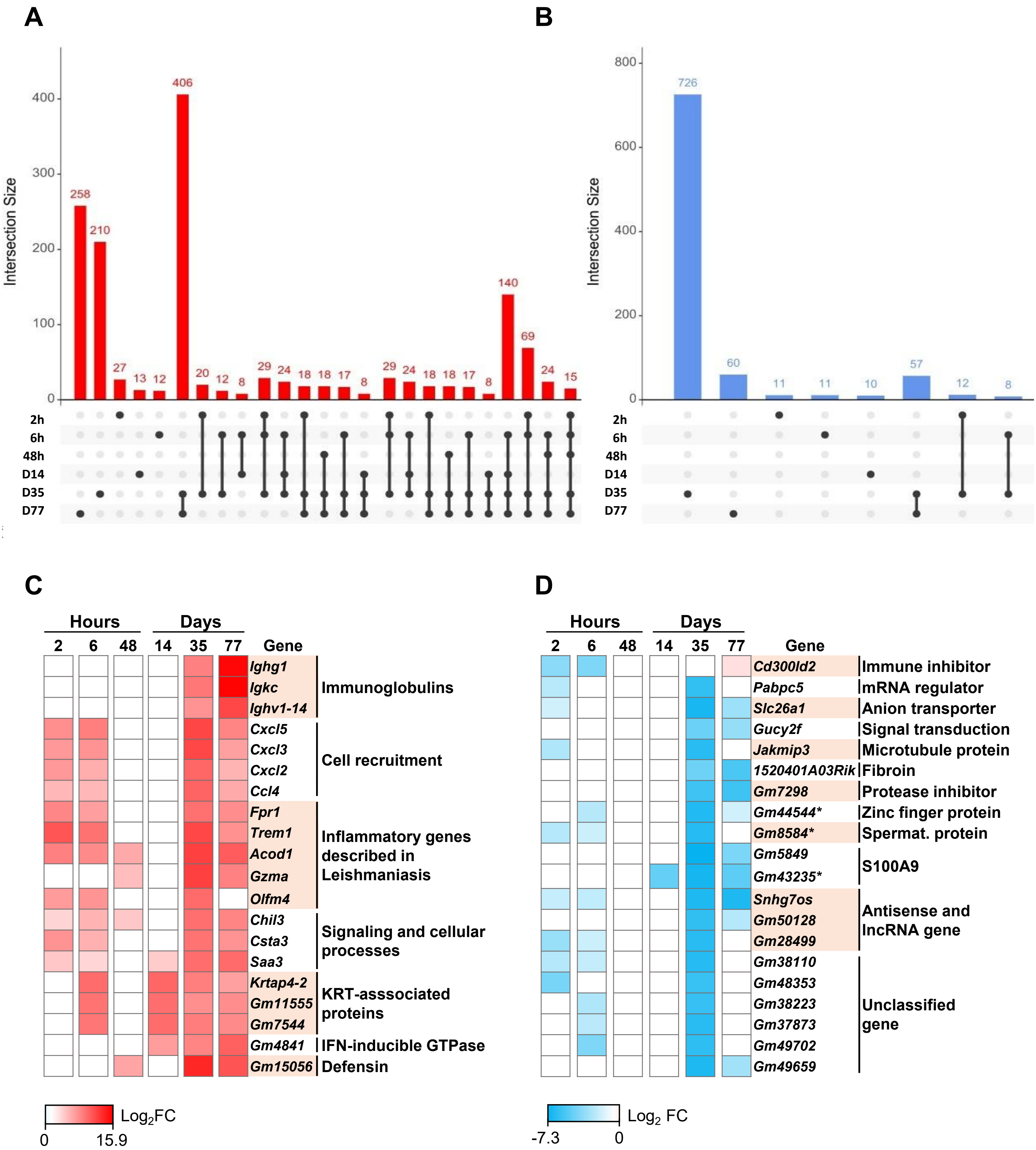

Supplementary Figure 4. Shared differentially expressed genes (DEGs) during lesion development and healing in murine cutaneous leishmaniasis. (A, B) UpSet plots showing shared upregulated (A) and downregulated (B) DEGs across time points. Dark dots in the matrix indicate intersections between time points, and the colored bars above indicate the number of DEGs shared in each intersection. Only genes with FDR ≤ 0.05 and absolute $\log_2$FC ≥ 2.0 were included. **(C, D)** Heatmaps displaying the top 20 upregulated (C) and downregulated (D) DEGs shared by at least two time points. Columns represent time points, and rows represent individual genes, color-coded by log2 fold-change relative to controls. Only genes with FDR ≤ 0.05 were considered for analysis. Asterisk (*) denotes pseudogenes.

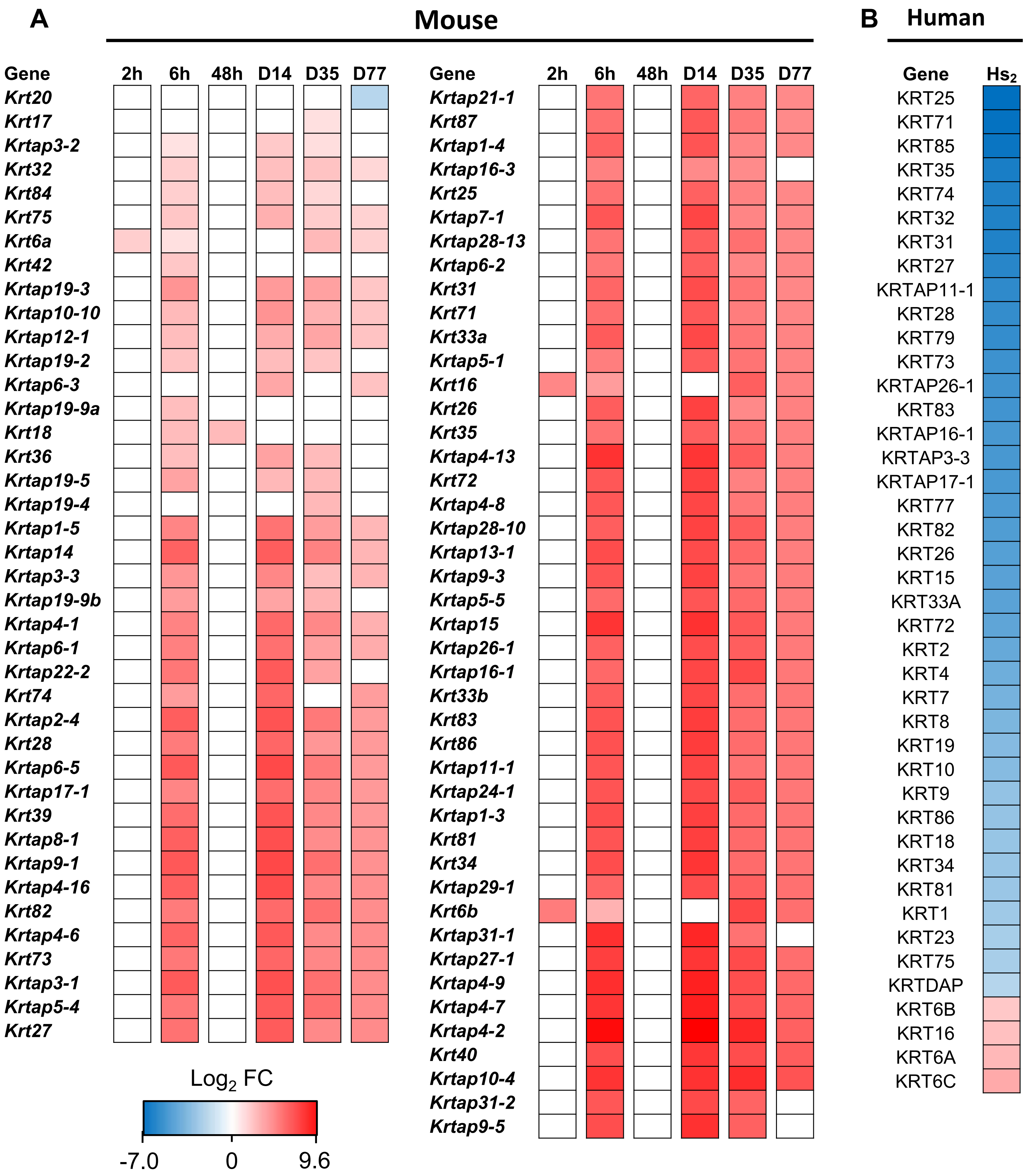

Supplementary Figure 5. Expression profile of tissue remodeling genes in murine and human cutaneous leishmaniasis. (A) Heatmap of keratin-associated genes in *L. braziliensis*-infected mice. Gene names are shown on the left, and the columns represent experimental time points. The color scale (blue to red) indicates fold changes relative to controls, with FDR ≤ 0.05. **(B)** Corresponding human data from Christensen et al. (2016) (*Hs_2*).

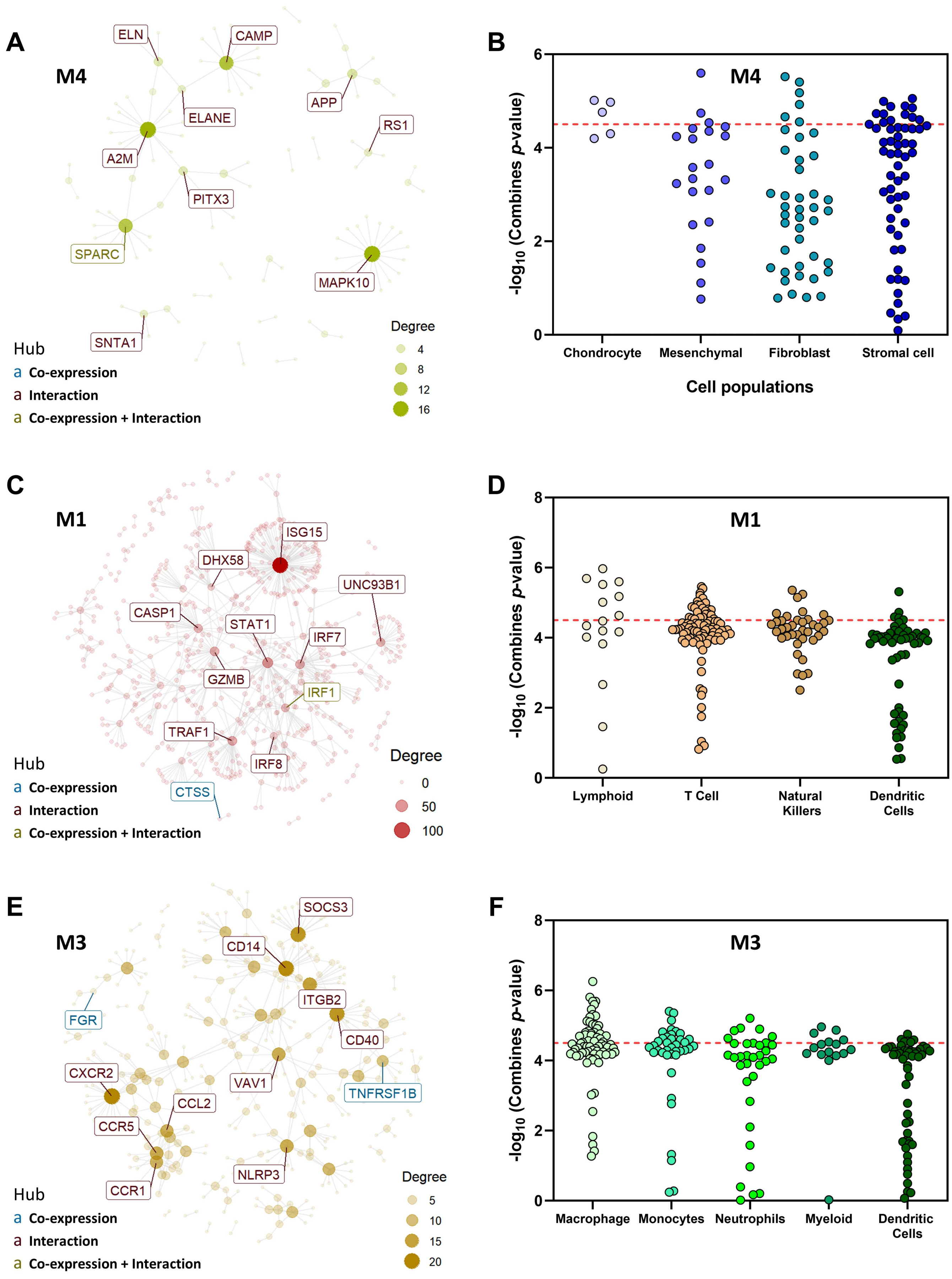

Supplementary Figure 6. Gene co-expression networks and cell type prediction from lesion-associated modules identified by CEMiTool. (A, C, E) Co-expression networks for modules M4 (A), M1 (C), and M3 (E), highlighting the most connected (hub) genes. Node size is proportional to the degree of connectivity. (B, D, F) Cell type prediction for modules M4 (B), M1 (D), and M3 (F) using WebCSEA. The red line indicates the Bonferroni-corrected significance threshold. Combined p-values, calculated using Fisher's method, represent the integration of trait-associated genes and cell types within the WebCSEA database, and reflect the tissue and cell-type specificity of each module.

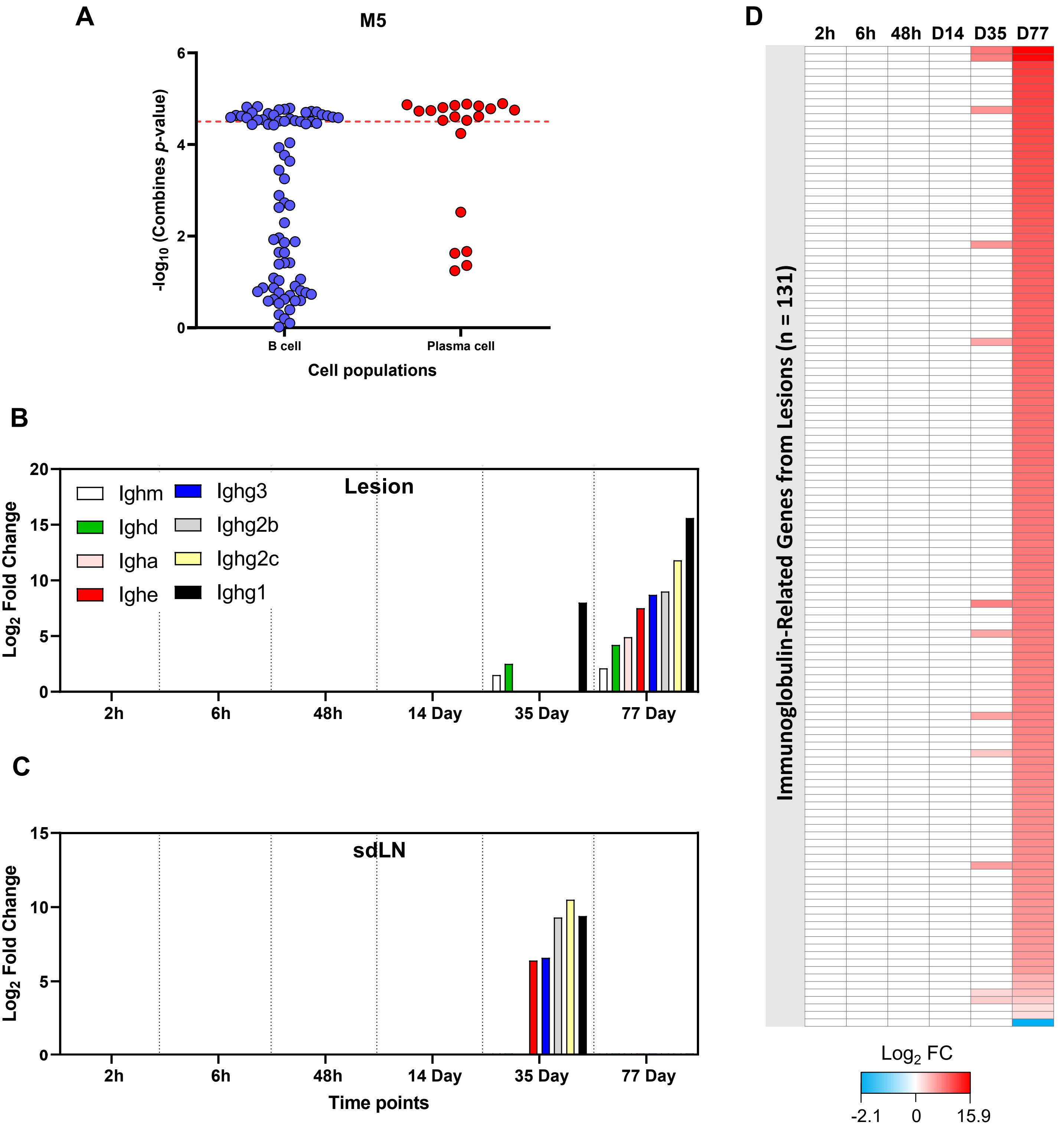

Supplementary Figure 7. Immunoglobulin gene expression dynamics in lesions and dLN during murine cutaneous leishmaniasis. (A) Cell type prediction using genes from modules M5 (lesion) via WebCSEA. The red line indicates the Bonferroni-corrected significance threshold. Dots represent combined p-values of trait-associated genes across cell types within the WebCSEA database, calculated using Fisher's method to indicate tissue and cell-type specificity. **(B-C)** Temporal expression profiles of immunoglobulin genes in lesions **(B)** and dLNs **(C)** throughout infection. Colored bars indicate fold changes in gene expression at each time point (2 h, 6 h, and 48 h, and Days 14, 35, and 77). **(D)** Heatmap showing expression levels of immunoglobulin-related genes in lesions across time points. Columns represent time points, and rows represent individual genes. The color scale reflects log2 fold-changes in gene expression, with FDR ≤ 0.05.

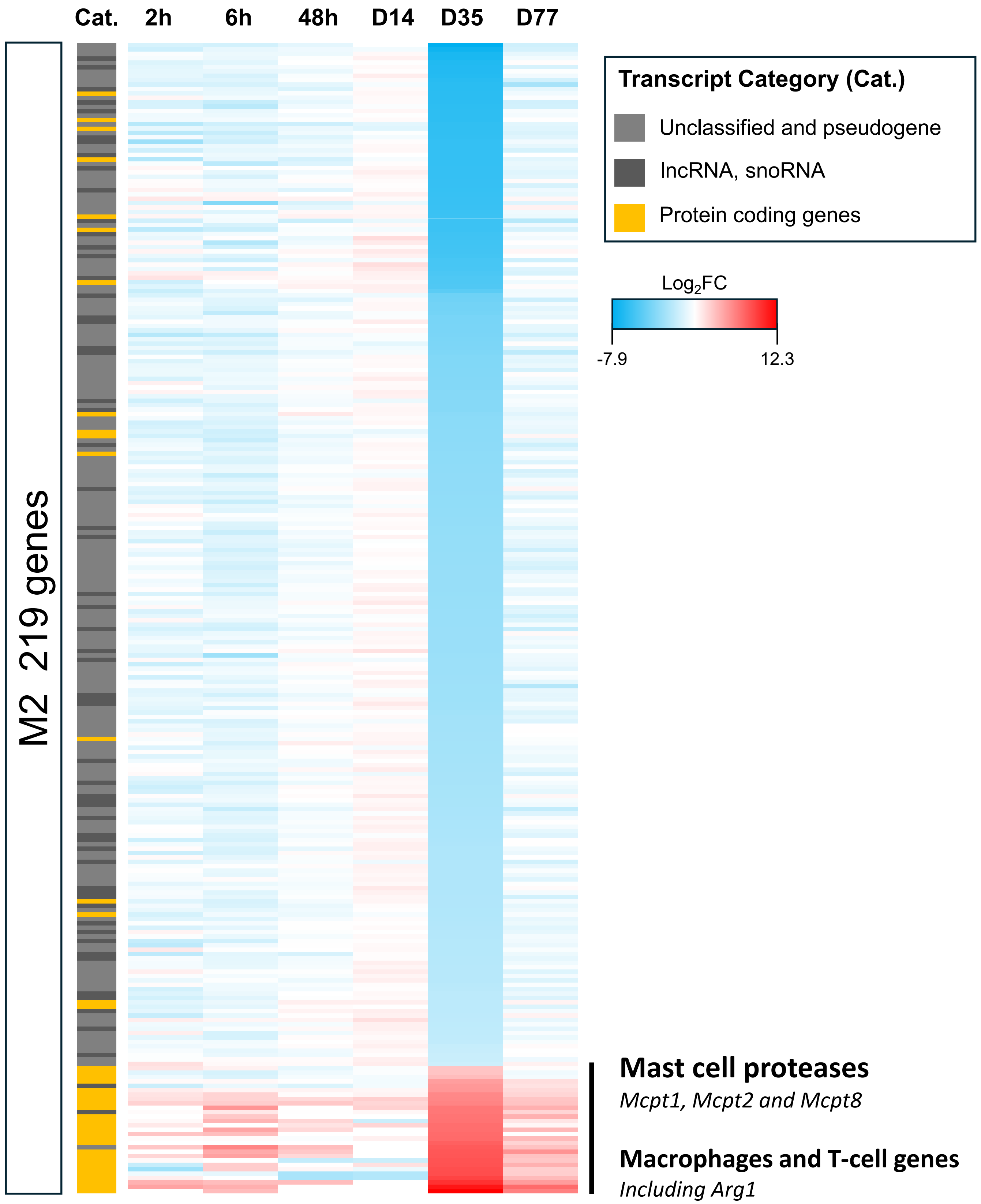

Supplementary Figure 8. Gene expression profile of module 2 (M2). Heatmap showing the temporal expression patterns of genes within module 2. On the left, genes are categorized as unclassified/pseudogenes, lncRNA/snoRNA, or protein-coding. Columns represent time points, and rows represent individual genes. The color scale reflects log2 fold changes in expression relative to controls, with FDR ≤ 0.05.

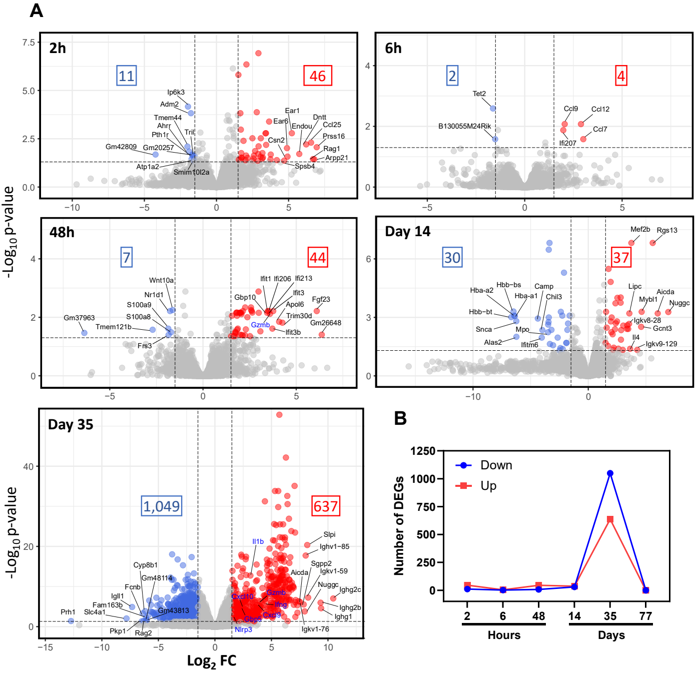

Supplementary Figure 9. Transcriptomic signatures of draining lymph nodes during the course of murine *L. braziliensis* infection. (A) Volcano plots showing differentially expressed genes (DEGs) at 2, 6, 48 hours, and at 14 and 35 days post-infection. Red dots indicate upregulated DEGs ($\log_2$FC > 1.5, FDR < 0.05), and blue dots represent downregulated DEGs ($\log_2$FC < −1.5, FDR < 0.05). Fold changes were calculated relative to non-manipulated controls. Genes labeled in blue are associated with the putative "metapathway" described by Novais et al. (2015). The number of up- and downregulated genes at each time point is shown in the upper right and upper left corners of each plot, respectively and summarized in the graph in **(B)**.

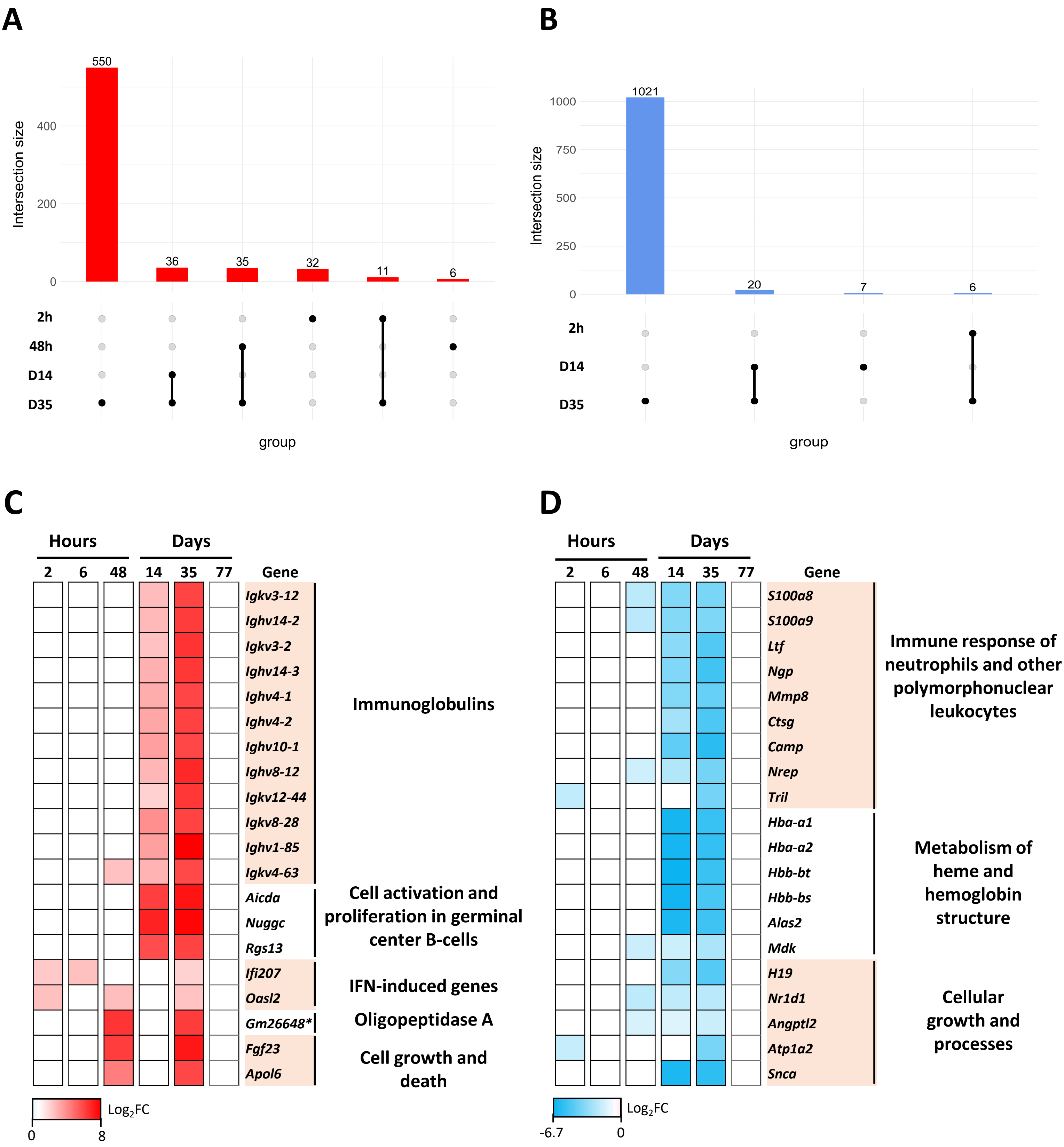

Supplementary Figure 10. Shared differentially expressed genes (DEGs) in draining lymph nodes (dLNs) during lesion development and healing in murine cutaneous leishmaniasis. (A, B) UpSet plots showing shared upregulated (A) and downregulated (B) DEGs across time points. Dark dots in the matrix indicate intersections between time points, and the colored bars above indicate the number of DEGs shared in each intersection. Only genes with FDR $\leq$ 0.05 and absolute log2FC $\geq$ 1.5 were included. (C, D) Heatmaps displaying the top 20 upregulated (C) and downregulated (D) DEGs shared by at least two time points. Columns represent time points, and rows represent individual genes, color-coded by log2 fold-change relative to controls. Only genes with FDR $\leq$ 0.05 were considered for analysis. Asterisk (*) denotes pseudogenes.

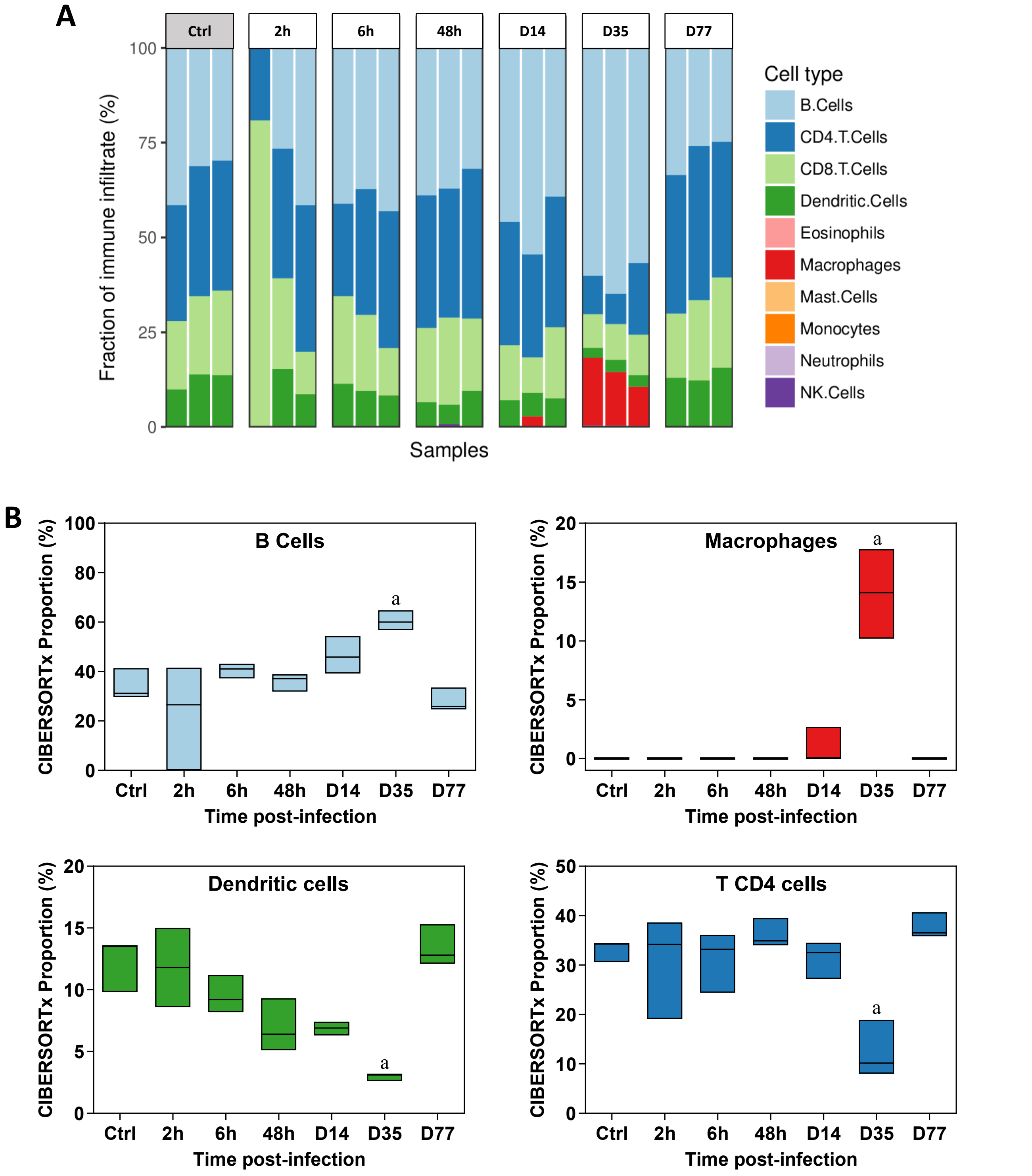

Supplementary Figure 11. Predicted cell composition of draining lymph nodes (dLNs) during *L. braziliensis* infection. (A) Bar plots showing the distribution of 10 immune cell types in each dLN sample across different time points, as estimated by CIBERSORTx. (B) Box plots displaying the predicted proportions of B cells, macrophages, dendritic cells, and $CD4^{+}$ T cells at each time point. Significant differences between groups were assessed by one-way ANOVA with Tukey's post-test.

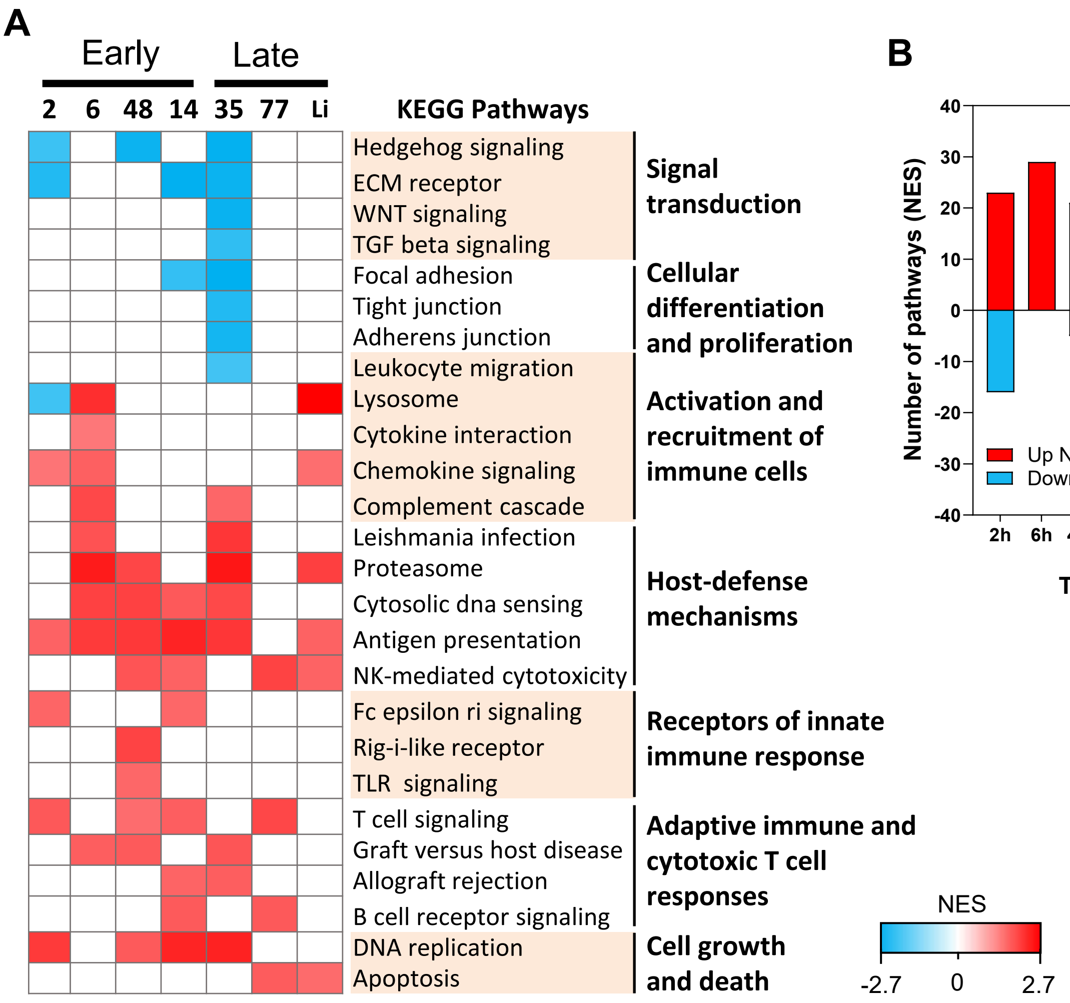

Supplementary Figure 12. Enrichment analysis of *Leishmania*-infected draining lymph nodes (dLNs). (A) Gene Set Enrichment Analysis (GSEA) results showing pathways enriched in dLNs, based on the KEGG pathway collection. Columns represent sample time points, with the final column corresponding to a canine *L. infantum* visceral infection dataset (*Li*). Rows represent pathways, with color intensity indicating activation or repression according to the normalized enrichment score (NES). Only pathways with FDR ≤ 0.05 were included. (B) Total number of KEGG pathways with positive or negative NES identified by GSEA, respectively.

3. References

1. Liu Z, Bian X, Luo L, Björklund ÅK, Li L, Zhang L, et al. Spatiotemporal single-cell roadmap of human skin wound healing. Cell Stem Cell. 2025;32: 479-498.e8. doi:10.1016/j.stem.2024.11.013

2. Reynolds G, Vegh P, Fletcher J, Poyner EFM, Stephenson E, Goh I, et al. Developmental cell programs are co-opted in inflammatory skin disease. Science. 2021;371. doi:10.1126/science.aba6500

3. McGinnis CS, Murrow LM, Gartner ZJ. DoubletFinder: Doublet Detection in Single-Cell RNA Sequencing Data Using Artificial Nearest Neighbors. Cell Syst. 2019;8: 329-337.e4. doi:10.1016/j.cels.2019.03.003

4. Hao Y, Stuart T, Kowalski MH, Choudhary S, Hoffman P, Hartman A, et al. Dictionary learning for integrative, multimodal and scalable single-cell analysis. Nat Biotechnol. 2024;42: 293–304. doi:10.1038/s41587-023-01767-y

5. Korsunsky I, Millard N, Fan J, Slowikowski K, Zhang F, Wei K, et al. Fast, sensitive and accurate integration of single-cell data with Harmony. Nat Methods. 2019;16: 1289–1296. doi:10.1038/s41592-019-0619-0

6. Blondel VD, Guillaume J-L, Lambiotte R, Lefebvre E. Fast unfolding of communities in large networks. J Stat Mech Theory Exp. 2008;2008: P10008. doi:10.1088/1742-5468/2008/10/P10008

7. Farias Amorim C, Lovins VM, Singh TP, Novais FO, Harris JC, Lago AS, et al. Multiomic profiling of cutaneous leishmaniasis infections reveals microbiota-driven mechanisms underlying disease severity. Sci Transl Med. 2023;15. doi:10.1126/scitranslmed.adh1469

8. Chen S, Zhou Y, Chen Y, Gu J. fastp: an ultra-fast all-in-one FASTQ preprocessor. Bioinformatics. 2018;34: i884–i890. doi:10.1093/bioinformatics/bty560

9. Ewels P, Magnusson M, Lundin S, Käller M. MultiQC: summarize analysis results for multiple tools and samples in a single report. Bioinformatics. 2016;32: 3047–3048. doi:10.1093/bioinformatics/btw354

10. Dobin A, Davis CA, Schlesinger F, Drenkow J, Zaleski C, Jha S, et al. STAR: ultrafast universal RNA-seq aligner. Bioinformatics. 2013;29: 15–21. doi:10.1093/bioinformatics/bts635

11. Liao Y, Smyth GK, Shi W. featureCounts: an efficient general purpose program for assigning sequence reads to genomic features. Bioinformatics. 2014;30: 923–930. doi:10.1093/bioinformatics/btt656

12. Love MI, Huber W, Anders S. Moderated estimation of fold change and dispersion for RNA-seq data with DESeq2. Genome Biol. 2014;15: 550. doi:10.1186/s13059-014-0550-8

13. Wang X, Park J, Susztak K, Zhang NR, Li M. Bulk tissue cell type deconvolution with multi-subject single-cell expression reference. Nat Commun. 2019;10: 380. doi:10.1038/s41467-018-08023-x

14. Cai Y, Xiong M, Xin Z, Liu C, Ren J, Yang X, et al. Decoding aging-dependent regenerative decline across tissues at single-cell resolution. Cell Stem Cell. 2023;30: 1674-1691.e8. doi:10.1016/j.stem.2023.09.014
